# Acoel single-cell atlas reveals expression dynamics and heterogeneity of adult pluripotent stem cells

**DOI:** 10.1038/s41467-023-38016-4

**Published:** 2023-05-05

**Authors:** Ryan E. Hulett, Julian O. Kimura, D. Marcela Bolaños, Yi-Jyun Luo, Carlos Rivera-López, Lorenzo Ricci, Mansi Srivastava

**Affiliations:** 1 Department of Organismic and Evolutionary Biology, Museum of Comparative Zoology, Harvard University, Cambridge, MA 02138 USA; 2 Biodiversity Research Center, Academia Sinica, Taipei, Taiwan; 3 Department of Molecular and Cell Biology, Harvard University, Cambridge, MA 02138 USA

**Keywords:** Regeneration, Evolutionary developmental biology, Adult stem cells

## Abstract

Adult pluripotent stem cell (aPSC) populations underlie whole-body regeneration in many distantly-related animal lineages, but how the underlying cellular and molecular mechanisms compare across species is unknown. Here, we apply single-cell RNA sequencing to profile transcriptional cell states of the acoel worm *Hofstenia miamia* during postembryonic development and regeneration. We identify cell types shared across stages and their associated gene expression dynamics during regeneration. Functional studies confirm that the aPSCs, also known as neoblasts, are the source of differentiated cells and reveal transcription factors needed for differentiation. Subclustering of neoblasts recovers transcriptionally distinct subpopulations, the majority of which are likely specialized to differentiated lineages. One neoblast subset, showing enriched expression of the histone variant *H3.3*, appears to lack specialization. Altogether, the cell states identified in this study facilitate comparisons to other species and enable future studies of stem cell fate potentials.

## Abstract (web summary)

Multiple species can achieve whole-body regeneration through the action of adult stem cells, though little is known about how these cells compare across species. Here they investigated the stem cells of a marine worm, the acoel *Hofstenia miamia*, and identified heterogeneity of these cells during development and regeneration.

## Introduction

Regeneration is a fundamental biological process that, in most animal species, requires precise control of self-renewal and differentiation of stem cell populations or of dedifferentiated cells to achieve restoration of injured or lost tissue in the adult animal body. The phenomenon is widely observed across animal phyla, albeit with different animals displaying varying capacities to replace tissue^1^. In vertebrates, most post-embryonic stem cells are lineage-restricted, corresponding to limited regeneration capacity in the adult body. In contrast, many distantly-related invertebrates such as cnidarians, acoels, planarians, and tunicates can regrow their whole bodies after injury, and, as adults, carry a large pool of effectively pluripotent stem cells that enable regeneration of virtually any missing cell type^2^. Whether conserved or divergent mechanisms govern regulation of these stem cells across metazoans remains unclear. Thus, identification of mechanisms for the regulation of adult stem cells is central to a comprehensive understanding of whole-body regeneration.

Adult pluripotent stem cell (aPSC) populations identified in diverse invertebrates express homologs of the Piwi gene family and, in some cases, other well-known germline genes^3,4^. The planarian *Schmidtea mediterranea* can regenerate any missing tissue and has a large population of Piwi-expressing aPSCs called “neoblasts” that are required for regeneration^5,6^. Single neoblasts transplanted into irradiated animals expand clonally (thus referred to as clonogenic- or c-neoblasts), differentiate into all tissue types of the adult animal, and restore the regenerative capacity of their hosts^7^. However, neoblasts in adult worms are transcriptionally heterogeneous—a large proportion are likely lineage-specialized progenitors^8–15^ and it is unknown if any one subset of the total neoblast population is clonogenic. Recent studies suggest that these specialized neoblasts may be able to give rise to progeny with a different fate, i.e. seemingly lineage-restricted cells could be pluripotent^16^. The Piwi-expressing aPSCs of cnidarians, called i-cells, show slightly different potency in different species^17^. Within cnidarian systems, there is heterogeneity within the population of i-cells found in *Hydractinia* and *Hydra*^17^ and these stem cell populations are considered to be multipotent or effectively pluripotent^18–21^. While heterogeneity is emerging as a unifying theme for planarian and cnidarian aPSCs, precise comparisons of how these cells are regulated are lacking.

**Fig. 1:**
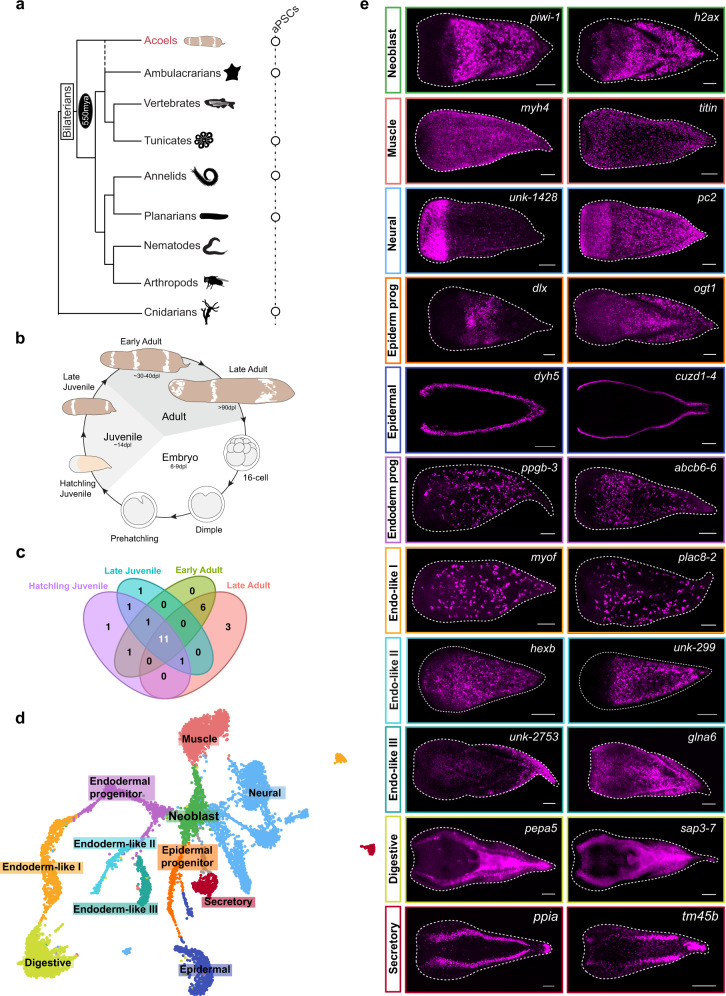
scRNA-seq reveals shared cell types present across postembryonic stages. **a** Schematic phylogeny of bilaterians with a cnidarian outgroup based on published literature^112^ highlighting the presence of adult pluripotent stem cells (aPSCs) across multiple metazoan lineages as well as the origin of bilateria ~550 million years ago (mya). Silhouettes of animals were modified from phylopic. Credit to B. Duygu Özpolot (tunicate and annelid) and Markus A. Grohme (planarian). **b** Life cycle schematic of *Hofstenia miamia*. *Hofstenia* embryogenesis takes about 8–9 days, and postembryonic development until sexual maturity (animals are hermaphroditic) takes about 6 weeks. The postembryonic stages are divided into 4 distinct stages: hatchling juvenile, late juvenile, early adult, and late adult. **c** Venn diagram of *Hofstenia* cell types detected across different postembryonic stages. There are 11 cell types that are consistent across postembryonic development. **d** Uniform Manifold Approximation and Projection (UMAP) representation of the hatchling juvenile scRNA-seq data set with the 11 cell types that were found consistent across stages. Gray clusters represent cell types that were not consistent across stages. **e** Fluorescent in situ hybridization of selected markers corresponding to each of the 11 shared identified cell types. Scale bars 100 µm.

Acoel worms are another example of an animal group that regenerate using neoblast-like aPSCs^22,23^ (Fig. 1a). Acoels hold a unique phylogenetic position that is sister to other bilaterians^24–31^ or to ambulacrarians^32–36^ that can inform stem cell differentiation dynamics and provide a comparative perspective to reveal the evolution of bilaterian cell-type programs. Here we applied single-cell RNA-sequencing (scRNA-seq) to profile cell states during different stages of postembryonic development and whole-body regeneration in the acoel *Hofstenia miamia*. *Hofstenia* regenerate robustly and are amenable to functional studies of regeneration. Despite the large evolutionary distance between *Hofstenia* and planarians, *Hofstenia* also have aPSCs called neoblasts that are the only proliferating somatic cells, are required for regeneration, and express homologs of Piwi^22,37^.

Here, we construct a single-cell atlas for *Hofstenia* and identify major cell types as well as the transcription factors required for their differentiation from stem cells. scRNA-seq of regenerating worms reveals that stem cells and differentiated cell types respond to amputation with dynamic but cell-type specific changes in gene expression. Further, we find that the aPSC population is composed of transcriptionally distinct subtypes that also respond differently to amputation, including an unspecialized neoblast subpopulation that putatively represents a pluripotent state.

## Results

### Cell types shared across juvenile and adult worms

*Hofstenia* are maintained in the laboratory as a sexually reproducing population of hermaphroditic adults. Zygotes undergo embryonic development and hatch out as juvenile worms in about 8–9 days, with post-embryonic development into sexually mature adult worms occurring over ~6 weeks^38^ (Fig. 1b). To facilitate a thorough characterization of cell types and their dynamics, we applied single-cell RNA-sequencing (scRNA-seq) using the InDrops platform^39,40^ in whole worms at four distinct stages of post-embryonic development: (1) hatchling juveniles, worms that have newly emerged from the egg shell upon completion of embryonic development (~8 days post lay, dpl), (2) late juveniles, worms with clear anterior and posterior bands of cream-colored pigmentation on their dorsal sides (typically 14 dpl), (3) early adults, worms with three bands of cream-colored pigmentation and visible oocytes on their ventral sides (typically 30–40 dpl), and (4) late adults, worms with three pigmentation bands that are sexually mature and have begun to produce embryos (typically >90 dpl) (Fig. 1b, Supplementary Data 1).

Unsupervised clustering of data from each stage revealed variable numbers of cell clusters across post-embryonic development (Fig. S1a, Supplementary Data 2). To determine the identities of these putative cell types, we focused on clusters that were consistently detected across all stages. As a first step, we calculated Pearson’s correlation coefficients based on the transcriptional profiles for all cell clusters identified in the four postembryonic stages and conducted hierarchical clustering (Fig. S1b). We found distinct clades where cell clusters from different stages grouped together, suggesting the presence of cell types with similar transcriptional profiles across postembryonic stages. Next, by projecting the expression of the top marker genes in these shared clusters across all four atlases, we were able to identify 11 transcriptionally distinct major cell types that were consistently present during postembryonic development (Fig. 1c). Additionally, we merged the data from all four postembryonic stages and obtained an atlas where marker gene expression revealed clear correspondence of the 11 putative cell types across stages (Fig. S2a, b). We focused on these shared cell types with the ultimate objective of making robust, stage-independent inferences regarding differentiation trajectories (see results section *Identification of putative differentiation trajectories*). Using the hatchling juvenile data set as our proxy (Fig. 1d), we identified highly expressed genes (Supplementary Data 2), performed gene ontology (GO) enrichment analysis (Fig. S1c), and characterized mRNA expression by fluorescent in situ hybridization (FISH) for each cluster shared across stages (Fig. 1e, Fig. S1d). Genes that were recovered as cluster markers exhibited similar gene expression patterns, (for example *myoferlin* and *plac8-2*, which are markers of the endodermal-like I cluster were expressed in large mesenchymal cells with similar distribution in Fig. 1e) suggesting the cell clusters correspond to cell types.

We found five clusters corresponding to known tissue types in *Hofstenia*: neoblast (stem cell), muscle, neural, epidermal, and digestive cells (Fig. 1d,e, Fig. S1b–d). These cell types and their associated biological functions correspond to cell types identified in the single-cell sequencing data of another acoel species, *Isodiametra pulchra*, suggesting these cell types are shared across acoels^41^. Genes enriched in the neoblast cluster included the known neoblast marker *piwi-1*^22^, and similar to the expression pattern of that gene in *I. pulchra*, they lacked expression anteriorly and labeled cells with an even distribution from the midsection along the anterior-posterior axis, to the posterior-most region of *Hofstenia*. Neural markers were highly expressed in cells in the anterior as well as in subepidermal cells in the entire body, matching the patterns of known neural markers^42^. Epidermal marker gene expression was detectable in cells on the dorsal and ventral surfaces of animals. The musculature in *Hofstenia* is well-characterized^42,43^ and we could directly observe muscle fibers in gene expression studies of muscle-related cell cluster markers, such as *myh4* (Fig. 1e) and *tpm3* (Fig. S1d). Similarly, the markers of digestive cells were expressed in cells in the interior of the animal, resembling the pattern observed in previously-studied gut markers^38^. In further corroboration of these experimental validations of cell type identities, we also found that previously identified neoblast, neural, epidermal, muscle, and gut genes characterized in *Hofstenia* were also expressed in the corresponding cell clusters in our scRNA-seq data, supporting our cell type classifications (Supplementary Data 2). Additionally, GO terms enriched in these cell clusters were also consistent with these identities, e.g., muscle system process in muscle cells, synaptic vesicle transport/assembly in neurons, cilium organization in epidermal cells (which are ciliated in *Hofstenia*), and lipid metabolic process in gut cells (Fig. S1c).

Among the remaining cell clusters, we hypothesized that two cell clusters corresponded to lineage-specialized progenitor populations related to epidermal and endodermal populations because they showed expression of both differentiated cell type markers of these lineages and of neoblast markers (Fig. S1e). Given that bona fide lineage tracing is yet to be performed in adult *Hofstenia*, our use of the term ‘progenitor’ here refers to putative lineage-specialized progenitor populations, which are also referred to as specialized neoblasts in planarians^9^. The markers of one of the remaining clusters were expressed in large mesenchymal cells with a scattered distribution throughout the body. Although this cell cluster was distinct from digestive cells, we found that these mesenchymal cells showed overlap in gene expression with the digestive cell cluster (Fig. S1e). Thus, we hypothesize that this could be an endodermal cell type, related to gut tissue, and therefore we named it Endoderm-like I. The two remaining clusters also shared gene expression with digestive and Endoderm-like I cells, and hence we refer to them as Endoderm-like II and III, respectively. The eleventh cluster is likely a secretory cell type based on the expression of genes with molecular functions associated with secretion^44^.

### Dynamic cell types and gene expression

**Fig. 2:**
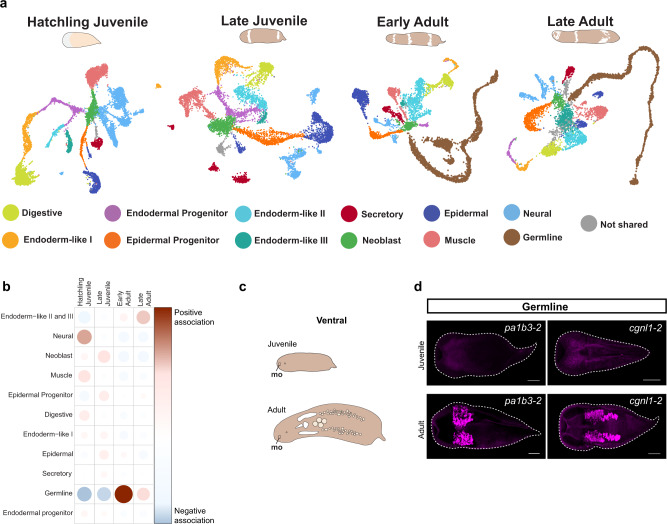
scRNA-seq highlights changes in gene expression of shared cell types and the appearance of germline cells during postembryonic development. **a** UMAPs representing shared cell types across postembryonic development. Transcriptionally similar clusters are denoted by the same color across stages. Gray clusters are not shared across stages. Cluster markers for each data set can be found in Supplementary Data 2. **b** Contingency table of Pearson residuals showing that the numbers of cells assigned to several major cell types are significantly different during postembryonic development (*p* = 2.2 × 10^−16^), with the largest difference attributed to the germline which is positively associated with early and late adult stages. **c** Schematics of the ventral view of juvenile and adult *Hofstenia*. In the adult, we have highlighted the putative reproductive structures associated with the germline; mo mouth. **d** FISH of germline markers *pa1b3-2* and *cgnl1-2* in both juveniles (top) and adults (bottom), with a striking lack of germline marker expression in the juveniles. Scale bars 100 µm in juveniles and 200 µm in adults.

Although the analyses of stage-specific atlases revealed strong correspondence of cell types across postembryonic developmental time, a few cell clusters were found to be stage-specific (Fig. 2a, Fig. S1a, Supplementary Data 2), including: hatchling juvenile cluster 16 (associated with endoplasmic reticulum function), late juvenile cluster 14 (associated with endoplasmic reticulum and protein folding), and late adult clusters 10 (associated with muscle), 22 (nervous system), and 29 (endocytosis) (Fig. S1a, Supplementary Data 2–3). The most notable cell type difference across the stage-specific atlases was the presence of germline-associated cell clusters in the early adult and late adult data sets relative to the data sets from juvenile worms (Fig. 2a, Fig. S1a, Fig. S2c, d). To quantitatively assess changes in cell type composition across stages, we focused on a merged data set (Fig. S2a, b, Supplementary Data 4). Applying a chi-squared test of independence, we found that the numbers of cells assigned to several major cell types were significantly different over the course of postembryonic development (*p* = 2.2 × 10^−16^). The contingency table of Pearson residuals showed that the most drastic difference could be attributed to the germline, with early and late adult stages having a positive correlation with the presence of germline cells (Fig. 2b). FISH for two germline markers in juvenile and adult worms corroborated this; no marker expression was observed in juveniles whereas adult worms showed clear expression in germline tissues (Fig. 2c, d).

In addition to considering cell number differences, we asked if shared cell types differed transcriptomically across stages. For each of the 11 shared cell types, we identified genes that were significantly differentially expressed in late juvenile, early adult, and late adult cells relative to cells in the hatchling juvenile (Supplementary Data 5–7). Gene Ontology (GO) analysis applied to the enriched genes showed a diversity of molecular functions and often corresponded to the functions of the cell type (Supplementary Data 5–7). For example, neuronal function terms were enriched in comparisons of neural cells and lipid metabolic processes were upregulated in digestive cells. Of note, in muscle cells, an upregulation of Wnt pathway ligands was observed in older postembryonic stages relative to in the hatchling juvenile. Given the known expression of Wnt ligands in this tissue in *Hofstenia* and their functions in patterning during regeneration and homeostasis^22,45,46^, future studies will be needed to assess if there is a functional significance to this increase level of Wnt gene expression.

### Identification of putative differentiation trajectories

Given that neoblasts are needed for the formation of new tissue in *Hofstenia*^22^, we next sought to identify the molecular signatures of this process in our scRNA-seq data. We found the topology of the Uniform Manifold Approximation and Projections (UMAPs) to be very consistent across the four postembryonic stages, always showing a branching structure radiating from a center. Specifically, neoblasts were located at the center, putative progenitor cells (namely the ones associated with endodermal and epidermal tissues) at the branch points, and mature cell types at the tips of the branches (Fig. 1d, Fig. S1a, Fig. S1c, Fig. S2a, b). We also found that while the clustering parameters did not recover distinct clusters for muscle and neural specialized neoblasts, cells with expression of both neoblast and differentiated cell markers were indeed present in our data set, often observable at the boundary between the neoblast cluster and the muscle/neural clusters. Although dimensionality reduction approaches such as UMAP do not show transcriptional trajectories of cells, and the topology is not evidence of a trajectory^47^, our observations of the UMAP topology were suggestive of putative differentiation trajectories in *Hofstenia*. We sought to rigorously test this hypothesis, and therefore we utilized URD^48^ for trajectory inference and to identify genetic effectors of cellular differentiation.

**Fig. 3:**
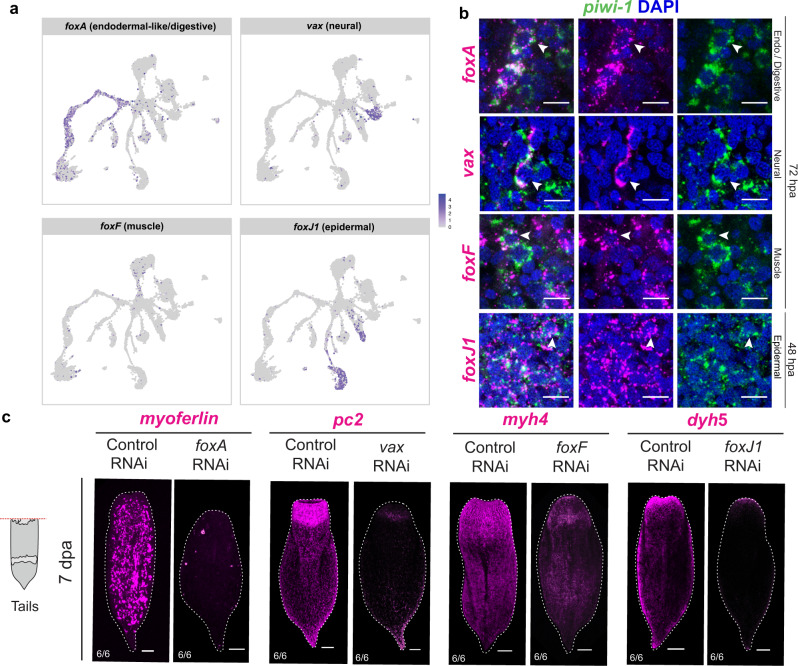
Transcription factors expressed in putative specialized neoblast populations are required for the differentiation of major cell types. **a** UMAP projection of transcription factors expressed in putative differentiation trajectories from URD analysis. All transcription factors identified were found to be expressed in their corresponding differentiated cell types and/or putative specialized neoblast populations. **b** Double FISH of the candidate transcription factors with the stem cell marker *piwi-1* during regeneration for putative endodermal-like/digestive, neural, muscle, and epidermal specialized neoblasts. Co-expression of *piwi-1* and candidate transcription factors (denoted by white arrowheads) was detected at 72 hours post amputation (hpa) for putative endodermal/digestive, neural, and muscle specialized neoblasts while putative epidermal specialized neoblasts were detected at 48 hpa. Scale bars 10 µm. **c** RNAi of putative specialized neoblast transcription factor leads to loss of differentiated cell type expression during regeneration. Regenerating tail fragments are shown 7 days post amputation (dpa) and corresponding head fragments are in Fig. S3e. Scale bars 100 µm. Schematic of regenerating *Hofstenia* tail in (**c**) reprinted from Cell Reports, Volume 32, Issue 9, Ramirez, A.N., Loubet-Senear, K., and Srivastava, M., A Regulatory Program for Initiation of Wnt Signaling during Posterior Regeneration, 108098, Copyright (2020), with permission from Elsevier.

We focused on the hatchling juvenile stage data set, setting the neoblast cluster as the root, and clusters with terminally differentiated cell types as tips. The placement of cells along the branches of this tree can be treated as a hypothesis of differentiation trajectories. To identify putative regulators of these differentiation trajectories, we identified genes that: (1) encoded transcription factors (TFs), (2) were significantly enriched in branch points, and (3) had homologs with known functions in differentiation of tissues in other research organisms. Using this approach, we identified candidate regulators for muscle (*foxF* and *six1*), neural (*vax* and *nkx2-1*), epidermal (*foxJ1* and *dlx*), and digestive/endodermal (*foxA, ikzf-1)* cell differentiation (Fig. 3a, Fig. S3a, Fig. S3g, Supplementary Data 8). We found that these TFs were expressed either in the differentiated cell clusters and/or in specialized neoblasts (Fig. 3a, Fig. S3a). The two cell clusters we had hypothesized to be putative lineage-primed progenitors for the epidermal and endodermal lineages were placed in close relationship to differentiated epidermal and digestive cells, respectively, in the URD tree, corroborating our hypothesis from clustering of the scRNA-seq data (Fig. S3b).

We reasoned that if these TFs are expressed in specialized neoblasts that are in transition to acquiring a terminally differentiated identity, (1) their expression should be detectable in *piwi-1*^+^ cells (neoblasts), and (2) disrupting their transcripts should result in the elimination of corresponding differentiated tissues. We found that *piwi-1*^+^/TF^+^ cells were detectable starting at 72 hours post amputation (hpa) for most TFs, and at 48 hpa for *foxJ1* (Fig. 3b and Fig. S3c) for the epidermal lineage. We were also able to detect most *piwi-1*^+^/TF^+^ cells, albeit at lower numbers, in intact animals (Fig. S3c), suggesting that differentiation pathways in homeostasis mirror those in regeneration. We next conducted functional studies using RNA interference (RNAi) to determine whether these TFs were regulators of differentiation. We assessed this process during regeneration because amputating the animal forced cells to undergo differentiation to replace missing cell types and tissues. In seven out of eight gene knockdowns, animals formed unpigmented outgrowth (blastemas) and regenerated heads and tails, recapitulating wild type external morphology (Fig. S3d). Notably, *foxA* RNAi animals were the only ones that showed a visible phenotype with failure to regenerate head blastemas and pointed tails (Fig. S3d). FISH for markers of the corresponding differentiated cell types confirmed loss or reduction of endodermal/digestive (*myoferlin*), neural (*pc2*), muscle (*myh4*) and epidermal (*dyh5*) cells, respectively in *foxA, vax, foxF* and *foxJ1* RNAi animals (Fig. 3c, Fig. S3e). Additionally, the knockdowns for the other four transcription factors *(nkx2-1*, *six1*, *dlx*, and *ikzf-1*) resulted in a reduction of expression of the corresponding differentiated cell type markers relative to control RNAi (Fig. S3f). Together these data show that specific TFs that are expressed in subsets of *piwi-1*^+^ cells during regeneration and homeostasis are required for the regeneration of distinct differentiated tissues. Given that *piwi-1*^+^ cells are the only proliferative cells and are needed for regeneration in *Hofstenia*, this work uncovers the differentiation pathways required for the formation of new tissues from neoblasts.

### Cell type and gene expression dynamics during regeneration

**Fig. 4:**
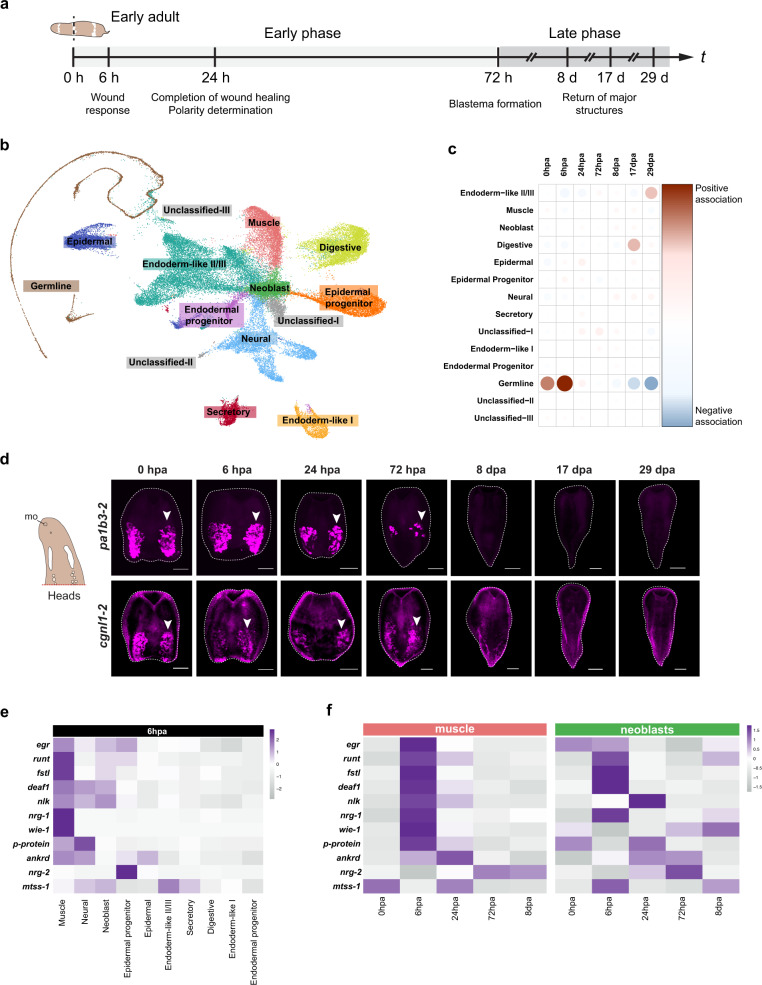
scRNA-seq during regeneration reveals cell-type specific responses. **a** *Hofstenia* regeneration highlighting time points sampled for scRNA-seq and associated events, with hours (h) and days (d) (*t* = time). **b** Merged regeneration UMAP of scRNA-seq data from the following time points: 0 hpa, 6 hpa, 24 hpa, 72 hpa, 8 dpa, 17 dpa, and 29 dpa. The 11 major cell types identified during postembryonic development are recapitulated here in the regeneration data, with neoblasts located in the center and differentiated cell types radiating out. **c** Contingency table of Pearson residuals showing that the numbers of cells assigned to several major cell types are significantly different during regeneration (*p* = 2.1 × 10^−16^), with the germline cells contributing to this by becoming progressively diminished over the course of the month following amputation showing a positive association with early timepoints and a negative association with later timepoints. **d** Left: Schematic of ventral view of adult head fragment with putative reproductive structures in white, mo mouth. Right: FISH of germline markers *pa1b3-2* (top) and *cgnl1-2* (bottom) in regenerating adult head fragments showing the gradual loss of germline-associated gene expression (white arrowheads) during regeneration over the course of 29 days. Scale bars 200 µm. Associated tail fragments in Fig. S4e. **e** Cell-type specific expression of EGR-GRN at 6 hpa. Cells from the merged regeneration data were subsetted to focus on the 6 hpa population and a heatmap depicting average expression was generated. All major cell-types were populated with cells from 6 hpa. **f** Dynamic expression of EGR-GRN members reveals temporal cell-type specific responses during regeneration. Heatmap of the muscle population shows dynamic expression of nearly every EGR-GRN member at 6 hpa. Heatmap of neoblast population show subsets of the EGR-GRN expressed at both 6 hpa and 24 hpa.

With single-cell transcriptional profiles for major cell types in hand, we next sought to compare cell type and gene expression dynamics during regeneration. In order to evaluate cell-type specific responses to wounding, we generated scRNA-seq data from combined regenerating head and tail fragments from early adult worms at seven timepoints (0, 6, 24, and 72 hpa, and 8, 17, and 29 days post amputation, dpa) (Fig. 4a). These time points were selected to capture major events during *Hofstenia* regeneration, including the wound response (6 hpa), completion of wound healing and polarity determination (24 hpa), blastema formation (72 hpa), and the return of most major structures during regeneration such as the brain (8 dpa)^22,42,46,49^. 8 dpa is the typical end-point of regeneration assays in acoels and planarians^42,50–53^, and we added the later 17 dpa and 29 dpa time points, to ask if these data would recover a signature for the “end” of regeneration. In other words, to see if gene expression would return to steady state a month after amputation. The 0 hpa data set serves as a control, capturing the profile of cells right at the moment the worms were amputated.

Unsupervised clustering of a data set that merged all regeneration time points recovered the major cell types identified in the postembryonic stages, including germline clusters, with the UMAP showing similar structure—neoblasts at the center and differentiated cell types at the periphery (Fig. 4b, Fig. S4a, Supplementary Data 9). Each cluster within the UMAP was composed of cells from each of the regeneration time points, with no clearly identifiable regeneration-specific cluster (Fig. S4b–d). A chi-squared test of the composition of each cluster by stage showed a significant change in cluster-specific cell numbers over the course of regeneration (*p* = 2.1 × 10^−16^), and the contingency table of Pearson residuals revealed germline cells to be the major contributor to these compositional differences (Fig. 4c). Specifically, this analysis showed that whereas the worms started out with many germline cells, the numbers of these cells became progressively diminished over the course of the month following amputation (Fig. S4b). FISH studies of germline marker genes in regenerating early adult worms corroborated this finding (Fig. 4d and Fig. S4e).

Next, we assessed how the major cell types, which are all present in all stages of regeneration, respond to amputation. We identified genes that showed significant upregulation over the course of regeneration within each cell type cluster in the merged data set (Supplementary Data 10–20). Many significant differences were recovered in the 29 dpa data set relative to 0 hpa, suggesting that in this experiment, the cells had not returned to original states one month post amputation. Our data were most informative for the immediate response to amputation (6 hpa vs. 0 hpa), which we found differs between tissues in terms of numbers and types of genes affected. Among differentiated cell types, digestive and epidermal cells showed significant upregulation of scores of genes, many corresponding to catabolic processes; in contrast, muscle and neural cells showed upregulation of <20 genes (Supplementary Data 10–13). Strikingly, the most significantly and highly upregulated gene in muscle cells at 6 hpa relative to 0 hpa was *egr*, a zinc finger transcription factor known to control a gene regulatory network (Egr-GRN) that is induced upon amputation in *Hofstenia*^49^. Other members of the Egr-GRN, such as *runt*, *fstl*, and *nlk* were also significantly upregulated in muscle cells by 6 hpa relative to 0 hpa. Although not all members of the Egr-GRN were recovered as significantly upregulated in our data set, we reasoned that this could be attributed to our experimental design—the single-cell data sets were derived from whole fragments, not just wound sites, which is where these genes are normally seen to be enriched^49^. We therefore next focused on relative levels of expression of Egr-GRN member genes (Fig. 4e, Fig. S4f).

The majority of genes in the Egr-GRN showed high expression in muscle cells at 6 hpa, a time point when GRN members show peak expression in bulk transcriptome data and in whole-mount gene expression studies^49^. Strikingly, the majority of endodermal cell types, including digestive cell types, showed very little expression of any genes in the Egr-GRN. Other cell types (neural, neoblast, epidermal progenitors, epidermal cells, and endodermal-like II/III, secretory) expressed some Egr-GRN members. Overall, it appears that muscle, neurons, epidermal progenitors, and neoblasts upregulate Egr-GRN members in response to injury, while endodermal tissues do not (Fig. 4e). It is important to note, however, that unbiased differential expression studies did reveal many other genes that are differentially upregulated in endodermal tissues, and future studies will decipher the functional importance of different wound response pathways in different cell types (Supplementary Data 14–16). Interestingly, some Egr-GRN members showed cell type specificity, e.g. *nrg-1* and *wie-1* were only highly expressed in muscle. These observations suggest that Egr is not equally wound-induced in all cell types, upregulating different genes in different tissues.

To identify temporal expression patterns of the Egr-GRN in specific cell types, we focused on muscle and neoblasts because our data showed significant upregulation of some Egr-GRN member genes in these cell populations (*egr*, *runt*, *fstl*, *nlk* in muscle; *fstl* in neoblasts). We subset the muscle and neoblast populations individually from the merged regeneration UMAP and looked at how the Egr-GRN member expression changed upon amputation up through 8 dpa (Fig. 4f). All but two GRN members (*nrg-2* and *mtss-1*) showed robust upregulation from 0 to 6 hpa in muscle, whereas *wie-1, p-protein* and *nrg-2* did not appear to be wound-induced in neoblasts. Of the Egr-GRN genes upregulated in both cell types, some including *runt*, *fstl*, *deaf1, nrg-1* peaked at 6 hpa while *ankrd* peaked at 24 hpa. *nlk*, which showed peak expression at 6 hpa in muscle, was upregulated more gradually in neoblasts, showing peak expression at 24 hpa. This suggests that, in addition to determining which target genes get activated, the cellular context may impact the temporal dynamics of their activation.

### *Hofstenia* neoblasts are transcriptionally heterogeneous

We noted that our data set did not show a substantial upregulation of *egr* in the neoblast cluster relative to the levels observed in other cell types such as in muscle (Fig. 4e, f and Fig. S4f), contrary to previously reported expression of *egr* mRNA in *piwi-1*^+^ cells in amputated worms^49^. Given the well-studied heterogeneity of neoblasts in planarians^13,54,55^, we asked whether heterogeneous induction of the Egr-GRN in specialized neoblast populations could underlie the lack of signal for wound-induced *egr* expression in our data set. Therefore, we next sought to identify heterogeneity within the neoblast population.

**Fig. 5:**
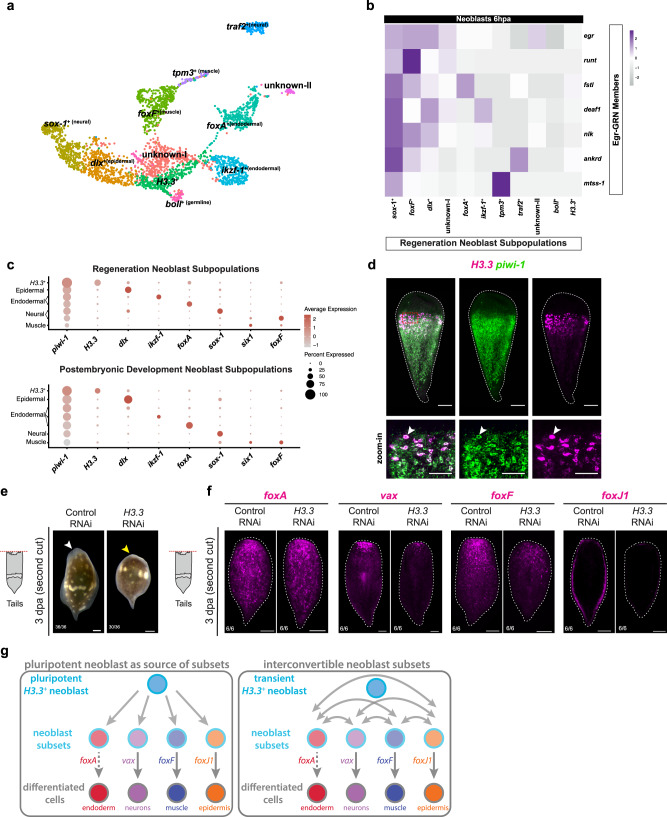
Neoblast subtype dynamics during postembryonic development and regeneration. **a** UMAP of neoblasts derived from the merged regeneration data set show subpopulations annotated with differentially expressed marker genes. **b** Heatmap of neoblast subpopulations at 6 hpa showing dynamic expression of EGR-GRN members. The *sox-1*^+^ neoblast subpopulation expresses every EGR-GRN member. Every other subpopulation expresses a suite of the EGR-GRN except for the *boll*^+^ which we hypothesize is a specialized germline neoblast and the *H3.3*^+^ subpopulation which shows downregulation of many members. **c** Dot plots of the neoblast subpopulations during both regeneration (above) and postembryonic development (below). Neoblast subpopulations that are consistently present in both the postembryonic and regeneration data sets are shown on the y-axis. Genes shown on the *x*-axis represent a major marker of each of these subpopulations. Strikingly, the *H3.3*^+^ neoblast subpopulation has high expression of *piwi-1*, relative to other specialized neoblast populations. **d** FISH reveals that *H3.3* (magenta) labels large mid-body cells that co-express (denoted by white arrowhead) *piwi-1* (green). Zoom-in region denoted by red dotted square. Scale bars 100 µm for 10x magnification and 50 µm for zoom-in. **e** RNAi of putative unspecialized neoblast subset marker, *H3.3*, leads to lack of blastema formation (denoted by yellow arrowhead) compared to proper blastema formation in control RNAi (denoted by white arrowhead). Regenerating tail fragments are shown 3 dpa after a second amputation and corresponding head fragments are in Fig. S5n. Scale bars 100 µm. **f** RNAi of putative unspecialized neoblast subset marker, *H3.3*, leads to a reduction in specialized neoblast transcription factor expression for *vax*, *foxF*, and *foxJ1*. *foxA* expression does not seem to be impacted. Regenerating tail fragments are shown 3 dpa after a second amputation. Scale bars 100 µm. **g** Schematic depicting the plausible scenarios where (i) *H3.3* labels a pluripotent neoblast population that acts as a source to distinct neoblast subsets which are lineage-primed progenitors giving rise to differentiated cell types or (ii) *H3.3* expression denotes a transient neoblast state and there are interconvertible neoblast subsets that are able to give rise to one another and then subsequently to differentiated cell types. Dashed arrow indicates that direct evidence of *foxA* control of endoderm differentiation was not found on this study. Schematic of regenerating *Hofstenia* tail in (**e**, **f**) reprinted from Cell Reports, Volume 32, Issue 9, Ramirez, A.N., Loubet-Senear, K., & Srivastava, M., A Regulatory Program for Initiation of Wnt Signaling during Posterior Regeneration, 108098, Copyright (2020), with permission from Elsevier.

We performed sub-clustering of the neoblast population from the merged regeneration data set to determine whether transcriptionally distinct subsets of neoblasts could be identified. We recovered 11 neoblast subpopulations, each with distinct transcriptional signatures (Fig. 5a, Supplementary Data 21). Seven of 11 clusters showed enriched expression of markers of differentiated types and/or of transcription factors we had identified as markers of specialized neoblasts. *tpm3*^+^ cells showed expression of markers associated with differentiated muscle cells. Based on functional studies of previously mentioned TFs, we identified four clusters we hypothesize represent epidermal (*dlx*^+^), muscle (*foxF*^+^), and endodermal (*ikzf-1*^+^ and *foxA*^+^) cells that likely represent lineage-specialized neoblasts. Two of the remaining clusters highly expressed *sox-1* and *traf2*, which showed enriched expression in cells connecting to the central neoblast cluster and to differentiated neurons in the postembryonic data set, indicating they may be neural specialized neoblasts (Fig. S5a). One cluster showed high expression of *boule-like* (*boll*), a gene known in other metazoan systems to be required for the specification of germ cells^56,57^. *boll* is highly expressed in putative germline cells in our early and late adult data sets, and we found it to mark two populations of cells that correspond to the location of germline in FISH studies of *Hofstenia* (Fig. S2c, d), therefore we inferred that *boll*^*+*^ cells are possibly specialized neoblasts that are primed to make the germline. One cluster, with a Histone H3 variant, *H3.3*, as its top marker gene did not express any genes corresponding to differentiated tissues. Two clusters failed to reveal any marker genes that showed specific expression in those cells when projected back onto the UMAP, and therefore we referred to them as unknown-I and unknown-II. Despite lacking markers with cluster-specific expression, these clusters did have genes with enriched expression relative to other neoblast subsets, with telomerase activity and DNA methylation biological processes being specific to the unknown-I cluster, and with the regulation of nitric-oxide synthase activity and Type B pancreatic cell development in the unknown-II cluster (Supplementary Data 21).

Next, we assessed the expression of the Egr-GRN members and found heterogeneous expression across these neoblast subclusters at 6 hpa (Fig. 5b). Three genes of the Egr-GRN, *nrg-2*, *nrg-1*, and *wie-1*, showed no or low expression in most cells across the neoblast subclusters, therefore we focused on the remaining ones (Fig. S5b). Strikingly, one subset, the *sox-1*^+^ cells (likely neural specialized neoblasts) showed robust expression of all other Egr-GRN genes (*egr*, *runt*, *fstl*, *deaf1*, *nlk*, *ankrd*, *mtss-1*). For two of these genes, *fstl* and *deaf1*, our data indicate that this expression at 6 hpa was a significant upregulation relative to 0 hpa, suggesting that the relative levels of expression of these genes could serve as a proxy for cell-type specific Egr-GRN-based wound responses (Supplementary Data 22). Several of these genes were also expressed in the *foxF*^+^ cells (likely muscle specialized neoblasts) and in the *dlx*^+^ cells (likely epidermal specialized neoblasts). Notably, one cluster, with *H3.3*^+^ cells, stood out as having very low expression of all GRN members at 6 hpa.

Given that the neoblast subsets showed heterogeneous responses during regeneration, we sought to gain a deeper understanding of these subtypes. We asked whether these subsets were unique to certain time points during regeneration and whether they could be identified in intact worms. To assess whether these subclusters were found across stages, during both regeneration and postembryonic development, we utilized hierarchical clustering and calculated the Pearson correlation coefficient and subclustering of the scRNA-seq data (Fig. S5c). We found neoblast subclusters associated with muscle (*foxF*^+^), neurons (*sox-1*^+^), epidermis (*dlx*^+^), and endoderm (*ikzf-1*^+^*, foxA*^+^) as well as *H3.3*^+^ cells present in multiple stages of regeneration and postembryonic development (Fig. 5a, Fig. S5c, d, f).

### Identification of a putatively unspecialized neoblast subset

Strikingly, *H3.3*^+^ neoblasts showed highest levels of *piwi-1* expression relative to all other specialized neoblast subsets that are found across post embryonic and regeneration time points (Fig. 5c). Given that the *H3.3*^+^ cluster is the only subset that is consistently present in all data sets from worms during postembryonic development and regeneration (Fig. 5a, Fig. S5c, d, f), remains unassigned to a lineage-specialized neoblast of differentiated tissue, is enriched with cells expressing genes associated with G2/M/S phase (Fig. S5e, g) and was the only neoblast cluster that failed to activate any Egr-GRN gene, we sought to characterize this marker and this cellular population further.

The putatively unspecialized *H3.3*^*+*^ cells showed enriched expression of two H3.3 variants, the top marker *H3.3* and its paralog *H3.3–2*, which belong to a family of H3.3 variants that includes four genes in *Hofstenia* (Fig. S5h). *H3.3* and *H3.3–2* contain a modified histone fold domain, AAIL. Neither *H3.3-3*, which contains the highly conserved AAIG motif in the histone fold, nor *H3.3-4*, which contains the divergent motif IALE, showed expression restricted to the neoblast population or were differentially expressed within the neoblast subpopulations (Fig. S5i). Therefore, we focused on *H3.3* and *H3.3–2* to understand the roles of Histone H3 variants in neoblast biology. Despite their high similarity of sequence at the amino acid level, these two genes are distinct at the nucleotide level (Fig. S5j), which allowed us to specifically investigate their expression and function.

High levels of *H3.3* transcripts were detectable in a population of large cells present in the midsection relative to the anterior-posterior axis of the worm in FISH experiments, with low levels detectable in cells with a neoblast-like distribution (Fig. 5d). In contrast, *H3.3–2* transcripts were more broadly expressed neoblast-like distribution, but lacking expression in large midbody cells (Fig. S5k). RNAi of *H3.3* and *H3.*3–2 did not impact regeneration upon one transverse amputation, however, the *H3.3* RNAi tail fragments failed to regenerate anterior tissues after a second round of double-stranded RNA (dsRNA) injection followed by amputation (Fig. 5e, Fig. S5l–o) while the *H3.3–2* RNAi tail fragments formed blastemas (Fig. S5n). Despite the similarities in nucleotide sequence of *H3.3* and *H3.3–2*, our experiments suggest specific targeting of *H3.3* (Fig. S5o). To determine the impact on regenerating tail fragments from the second round of *H3.3* RNAi where blastemas did not regenerate, we performed FISH to look at the regenerating nervous system and pharynx, two structures that are localized to the anterior of regenerating tail fragments by 7 dpa (Fig. S5p). The second round of RNAi impacted neural and pharyngeal regeneration in the *H3.3* RNAi condition, with a lack of clear gene expression associated with these two structures whereas worms under *H3.3–2* RNAi showed some expression associated with both the nervous system and the pharynx.

Double-FISH revealed that these *H3.3*^+^ cells also expressed *piwi-1*, corroborating *H3.3*^+^ cells as a bona fide subset of *piwi-1*^+^ cells (Fig. 5d). If the *H3.3*^+^ subset of neoblasts is specialized to a yet unknown differentiated lineage, we would expect abrogation of genes enriched in these cells not to impact the formation of other differentiated tissues. In the *H3.3* RNAi tail fragments with regeneration defects, some, but not all, markers of specialized neoblasts were impacted (Fig. 5f). Accordingly, we detected that *piwi-1*^*+*^ cells marked by *vax*, *foxF*, and *foxJ1, i.e*. neoblasts associated with neural, muscle, and epidermal lineages, respectively, were diminished in the *H3.3* RNAi animals relative to controls (Fig. S5q). These data suggest that *H3.3* may be needed for the formation of at least some specialized neoblasts.

This result could arise from two alternative scenarios: (1) *H3.3*^+^ cells could be a pluripotent subset that gives rise to specialized neoblasts, or, (2) *H3.3*^+^ cells could be a transient state that specialized neoblasts enter during self-renewal or differentiation (Fig. 5g). In both scenarios, *H3.3*^+^ cells emerge as representing an unspecialized state, which our experiments suggest is important for regeneration. The known roles of H3.3 variants in regulating stem cells in other systems like mouse and fly^58–64^ are consistent with this hypothesis. Notably, the *H3.3*^+^ cluster shows expression of certain transcription factors, including *FoxO* and *tbx* (Fig. S5r), which are also associated with pluripotent states in other systems^65–72^ and are found to be expressed in the embryonic lineage of neoblasts in *Hofstenia*^44^. Future studies using lineage tracing are needed to assess if *H3.3*^+^ cells are a source of some or all specialized neoblasts (notably, *foxA*^*+*^ endoderm-specialized neoblasts were not affected in our experiments), or if they are a transient state.

## Discussion

The single-cell transcriptome data sets analyzed here represent an experimentally-corroborated catalog of cell types during postembryonic development and regeneration in the acoel *Hofstenia miamia*. We found that major cell types were consistently identifiable throughout these processes and included differentiated cells such as muscle, neural, epidermal, and endodermal cells as well as aPSCs consisting of distinct neoblast subpopulations. We also provide functional validation of transcriptional regulators required for differentiation of neoblasts into terminally differentiated cell types. These data can provide insights into both the mechanisms of stem cell regulation and the evolution of cell type differentiation.

The transcriptome profiles for cell types and differentiation pathways in our data set provide an important point for comparison to study the evolution of major bilaterian cell types. Muscle, neural, and digestive cells in *Hofstenia* expressed homologs of well-characterized genes that are known to have important functions in these cell types in other bilaterians. For example, muscle cells express myosins and troponin, neurons express prohormone convertases and Trp channels, and gut cells express peptidases and cathepsins. However, the expression profiles of epidermal cells in *Hofstenia* did not reveal obvious shared genes for epidermal functions with the epidermal cells of other bilaterians found within the Nephrozoa. Previous work in the acoel *I. pulchra* identified similar cell types to those we found in *Hofstenia* based on enrichment of genes associated with biological functions^41^. In addition to uncovering these molecular functions in the corresponding *Hofstenia* cell types, our data set recovered transcription factors associated with these cell types. We noted that these transcription factors, needed for the formation of differentiated cell types in *Hofstenia*, were homologs of known regulators of these cell types during development and/or regeneration in other bilaterians. FoxF, a muscle regulator in *Hofstenia*, is also required for the regeneration of non-body wall muscle in planarians^73^. Six1 homologs have well established roles in muscle and muscle progenitor differentiation^74–77^, including during regeneration^78^. Vax and Nkx2 homologs, which are required for neural regeneration in *Hofstenia*, are well known neural TFs in bilaterians, and an Nkx2 homolog is needed for the maintenance of cholinergic, gabaergic, and octopaminergic neurons in the planarian central nervous system^79^. FoxJ is a regulator of ciliated epidermal cell types across eukaryotes, and specifically results in aberrant regeneration of the epidermis in planarians^80,81^, consistent with its role in regeneration of the *Hofstenia* epidermis. Dlx, another epidermal factor in *Hofstenia*, has homologs expressed in surface ectoderm across deuterostomes and is needed for differentiation of epidermal cells in vertebrates^82,83^. FoxA homologs are required for foregut/pharynx formation in many species^12,84–89^, and we found the FoxA homolog to be required for endodermal and digestive tissue in *Hofstenia*. Overall, differentiated cell types in *Hofstenia* appear to have clear molecular correspondence with their counterparts in bilaterians (except for epidermal cells), including in terms of the underlying transcriptional regulatory pathways for the formation of these tissues.

The single-cell atlas enabled us to probe the transcriptional wound response with cell-type resolution. We found that a major wound-induced GRN that is required for regeneration is upregulated in a subset of cells, with the most robust upregulation of the majority of GRN member genes occurring in muscle. This is consistent with observations of wound-induced gene expression in planarian muscle^90–92^ and of muscle being an important regulator of regeneration^45,93,94^. Other tissues showed upregulation of subsets of the GRN and also differed from muscle in the timing of upregulation of some genes, suggesting that the GRN has distinct dynamics and functions depending on the cellular context. We did not detect regeneration-specific clusters in our merged regeneration data sets but we did identify variable expression of the Egr-GRN across differentiated tissues, reminiscent of how post-mitotic cells respond to injury in the transient regeneration activated cells (TRACs) populations in *Schmidtea mediterranea*^95^. These data reiterate the importance of coordination across cell types to mediate whole-body regeneration and serve as an important resource for future work toward understanding these mechanisms.

Given the observation of *piwi*-expressing aPSCs in many distantly-related animals, our work, together with data from planarian neoblasts, suggests that we should expect aPSCs in other animals to be transcriptionally heterogeneous. To understand the evolution of this cell type, *piwi*-expressing aPSCs should be compared not as a monolithic type, but as a mixed population that may show nuanced differences across species. Notably, our data revealed one subset that was not assignable to a clear specialized neoblast type and showed distinct characteristics relative to other neoblast subsets. These *H3.3*^+^ cells, expressing a variant of histone H3, are a subset of *piwi-1*^+^ cells that do not upregulate wound response genes and show high *piwi-1* expression, and *H3.3* is required for the differentiation of neoblasts specialized for neural, muscle, and epidermal lineages. Whereas histone variants *H4*^96^ and *H2B*^97^ have been reported as enriched in planarian neoblasts, H3 variant roles are not fully-studied in planarian neoblasts^98,99^ or in cnidarian i-cells^100,101^. In *Hofstenia*, *H3.3*^+^ cells could represent a pluripotent state that is a predecessor to specialization or is a transient state of many specialized populations. Future functional work will reveal if the mechanism of *H3.3*^+^ action in these cells is similar to the known mechanisms of chromatin regulation by H3.3 in stem cells in other systems^102^. The data obtained in this study will also enable transgenesis-based tracing to assess the lineage relationships of neoblast subsets, specifically to test whether *H3.3*^+^ cells represent a pluripotent subpopulation.

## Methods

### Animal maintenance

Gravid adult worms were kept in plastic boxes (20–30 worms per 2.13-L Ziploc container) at 21 °C in artificial seawater (37 ppt, pH 7.9–8.0; Instant Ocean Sea Salt). Juvenile worms were kept in zebrafish tanks (~300 worms per tank). Seawater was replaced twice a week. Juvenile and adult worms were fed with rotifers *Brachionus plicatilis* and freshly hatched brine shrimp *Artemia* sp. twice a week, respectively. Juvenile worms were used for FISH and RNAi, except for the germline experiments which used adult worms. Animals are derived from the progeny of a randomly mating population of worms from Bermuda kept in laboratory culture since 2010.

### Cell dissociation and preparation

To avoid cell loss, worms were dissociated in calcium- and magnesium-free artificial seawater (CMF-ASW: 450 mM NaCl, 9 mM KCl, 30 mM Na_2_SO_4_, 2.5 mM NaHCO_3_, 25 mM HEPES, 10 mM Tris-HCl, 2.5 mM EDTA) in a 2-mL DNA LoBind tube (Eppendorf) by vigorously pipetting using a P1000 micropipette. Cell suspensions were passed through a 40-μm cell strainer (Falcon) to remove remaining aggregates. To further remove cell debris and cell-free RNA, cells were loaded on a BSA cushion (4% BSA in CMF-ASW) and pelleted at 500 × *g* for 5 min using a swing rotor. Cells were then washed twice with CMF-ASW by resuspension and spinning at 500 × *g* for 3 min. For inDrops encapsulation, cell suspensions in CMF-ASW were mixed 1:1 with 30% OptiPrep in CMF-ASW.

### inDrops encapsulation, library preparation, and RNA sequencing

Encapsulation of single cell suspensions was done at the Single Cell Core located at the Harvard Medical School using a custom inDrops microfluidics system^40^. *Hofstenia* is a marine organism, meaning it was necessary to keep the cells in seawater until the last possible moment before encapsulation to prevent cell death. We utilized a custom microfluidics chip offered at the core that allowed for the introduction of 1xPBS solution just prior to encapsulation. Cells were encapsulated on this chip and libraries of about 3000 cells were collected, with three libraries encapsulated per sample, totaling ~9,000 cells per sample/time point. Library preparation was performed by the Single Cell core as well. Library quality control via tapestation, qPCR quantification, and sequencing using the inDrops^40^ V3 design, on a NextSeq500 (Illumina) was performed by the Harvard Bauer Core. The following parameters were used for sequencing: read type = paired-end, Read 1 = 61 bp (transcript), Index Read 1 (i7) = 8 bp, Index Read 2 (i5) = 8 bp, and Read 2 = 14 bp.

### Single-cell analysis

Once reads were acquired, they were demultiplexed, mapped, and subsequently converted into count matrices using the methods described in the following github repositories: https://github.com/indrops/indrops and https://github.com/brianjohnhaas/indrops (which required python, bcl2fastq, RSEM, bowtie, samtools, pysam). Cell quality assessment, clustering, filtering, and dimensionality reduction was done using Seurat v3.1.4 in R^103–106^. Demultiplexing was performed using indrops.py v4.2. Reads from all libraries were pooled from each sample and were mapped to the *Hofstenia* transcriptome (NCBI Bioproject PRJNA512373) using Bowtie v1.1.1. Mapped reads were counted to generate a digital expression matrices using the script bam_to_count_matrix.pl. Data were imported into R and analyzed using Seurat 3.1.4. The analysis was conducted as described (http://satijalab.org/seurat). We determined that certain populations of the UMAP were either over- or under-clustered, based on known marker gene expression, depending on the different UMAP resolution parameters used in Seurat^106^. We iteratively altered parameters of clustering and UMAP rendering to make sure the results were robust and representative of the known biological cell types. Clustering was accomplished in an iterative manner, where we identified top marker genes and then projected back onto the UMAP plots to gauge specificity and uniformity of expression within their corresponding clusters. If we found top marker genes to be expressed highly in multiple clusters it suggested we were overclustering. If we found marker genes that were only expressed within a subset of the corresponding cluster, it suggested we were underclustering. To retain optimum cluster identities uniformly across the UMAP plot, we merged the cluster identities from different clustering parameters by using a custom python script on their metadata available on github: https://github.com/JulianKimura/Python_Scripts as “metadata_corr.py”. This merged metadata file was fed back into Seurat to generate the clusters that we found to most accurately represent the cell types in our data set. The code used to analyze the different data sets is available on our github repository: https://github.com/JulianKimura/Hulett_etal. Regeneration data sets and neoblast subsets were merged, without batch correction, as the samples were collected, encapsulated, and performed library preparation on them at the same time to minimize any noise to the data from batch effects. Summary statistics for scRNA-seq data sets can be found in Supplementary Data 5. Chi squared tests of independence were done on the postembryonic scRNA-seq and regeneration scRNA-seq data sets using the base R command to test the significance of cell types and their identities. We developed an online resource that allows for access and visualization of the scRNA-seq data, available at https://n2t.net/ark:/84478/d/q6fxc7jj^107^.

### Fixation and in situ hybridization

Whole worms and regenerating fragments were fixed in 4% paraformaldehyde in 1% phosphate-buffered saline with 0.1% Triton-X (PBST) for one hour at RT on nutator and stored in methanol at −20 °C until use. Digoxigenin and Fluorescein labeled riboprobes were synthesized as previously described^22^. Fluorescent in situ hybridizations (FISH) were performed following the protocol described in established protocols with minor modifications^22^. Animals were washed twice for ten minutes each in PBST and then blocked in a 10% horse serum in PBST for one hour at RT before antibody incubation. All washes the following day were made with PBST followed by tyramide with rhodamine or fluorescein development for 10 min. Detailed probe synthesis and in situ hybridization protocols with reagents and solution preparations were made following established protocols^22^. Primer sequences for screened genes provided in Supplementary Data 23.

### RNAi

Double-stranded RNA (dsRNA) synthesis was made following established protocols^22^. RNAi experiments were done by injecting the animals with dsRNA corresponding to the target gene into the gut for 3 consecutive days. dsRNA injections were performed using a Drummond Nanoject II. Animals were cut transversally at least 2 hours after the third injection and were allowed to regenerate for the appropriate number of days while being monitored for visible phenotypes or external defects. Control dsRNA for gene inhibition was the *unc22* sequence from *C. elegans* that is absent in *Hofstenia*. After the appropriate number of days post-amputation (dpa) animals were fixed and analyzed by FISH. Animals undergoing two rounds of injections and amputations were injected and soaked with dsRNA for 3 days and then amputated. Fragments were then injected for 3 more days (corresponding to days 5,6,7 post first amputation) and then amputated again. Fragments were allowed to regenerate for 3 days and then fixed.

### Gene ontology enrichment analysis

Gene ontology (GO) enrichment analysis was done using established methods as previously described^38^, where differentially expressed genes that showed significant upregulation (adjusted *p*-value <0.05) were used to generate the GO terms. The *Hofstenia* transcriptome was annotated with the best BLAST hits by e-value against human genes. The resulting lists of genes generated through the default differential expression analyses in Seurat (Wilcoxon Rank Sum Test) were used as input into the DAVID functional annotation tool. The representative GO term that was statistically significantly enriched was used to annotate our cell type atlas.

### Lineage reconstruction

Lineage reconstruction was done using the R package URD^48^. To construct lineage trees at the Hatchling Juvenile stage, we set the neoblast cluster as the root, differentiated cell types (e.g. digestive, epidermal, neural, muscle) as the lineage tree tips, and all other cells as the intermediate cells. Differential expression analysis using the AUPRC (area under the precision recall curve) test was performed to identify genes enriched in trajectories. All of the parameters utilized for constructing the URD tree along with differential expression are available on our github repository: https://github.com/JulianKimura/Hulett_etal.

### Pseudo-bulk heatmap

Cellular populations were subsetted from merged UMAPs based on clustering (i.e. the muscle cluster was extracted and further analyzed). Each cell population used in the pseudo-bulk heatmap were subsetted as its own Seurat object. The count matrices were then extracted and merged together in a bulk-RNAseq count matrix format where the rows contained gene names and the columns contained separate cell populations defined through clustering on Seurat. This count matrix was used as input into R and the Pearson’s correlation as well as heatmap generation was performed using the pheatmap package and the base R command “cor”. Metadata associated with each data set, including postembryonic development stage and regeneration time point were used to subset and further characterize expression dynamics.

### Phylogenetic analysis

Transcription factors identified from the scRNA-seq data, URD trajectory inference, and whose function were subsequently tested were assigned orthology. First, a BLAST was performed to putatively identify transcription factors of interest. To assign orthology, phylogenetic trees were constructed. Sequences were aligned with MUSCLE (v3.8.31)^108^. Alignments were trimmed using Gblocks^109,110^ with the least stringent parameters. Phylogenetic trees were inferred using Maximum Likelihood analysis with 1000 bootstrap replicates, implemented in RAxML (v8.2.4)^111^ using the WAG + G model of protein evolution.

### Statistics and reproducibility

Each FISH and RNAi experiment was performed in triplicate with at least 5 animals per condition. Sample sizes for the inDrops assay states 3000 cells/library. Each sequencing experiment was done in triplicate (at least). Differentially expressed genes between clusters (and specific populations of cells) was performed in Seurat using the non-parametric Wilcoxon rank-sum test. Adjusted *p*-values were calculated using Bonferroni correction.

### Reporting summary

Further information on research design is available in the Nature Portfolio Reporting Summary linked to this article.

## Supplementary information

Supplementary Information

Description of Additional Supplementary Files

Supplementary Data 1

Supplementary Data 2

Supplementary Data 3

Supplementary Data 4

Supplementary Data 5

Supplementary Data 6

Supplementary Data 7

Supplementary Data 8

Supplementary Data 9

Supplementary Data 10

Supplementary Data 11

Supplementary Data 12

Supplementary Data 13

Supplementary Data 14

Supplementary Data 15

Supplementary Data 16

Supplementary Data 17

Supplementary Data 18

Supplementary Data 19

Supplementary Data 20

Supplementary Data 21

Supplementary Data 22

Supplementary Data 23

Reporting Summary

## Data Availability

The sequencing raw reads and data generated in this study have been deposited in the NCBI BioProject database under accession codes PRJNA888438 (postembryonic development) and PRJNA908236 (regeneration time course). All processed matrices and R scripts used are available on github at https://github.com/JulianKimura/Hulett_etal (Zenodo 10.5281/zenodo.7700424). An online resource was generated to access and visualize the scRNA-seq, found at https://n2t.net/ark:/84478/d/q6fxc7jj.

## Code availability

Github web link for python scripts: https://github.com/JulianKimura/Python_Scripts.
