# Supplementary material for: Acoel single-cell atlas reveals expression dynamics and heterogeneity of adult pluripotent stem cells
Source: Nat Commun. 2023 May 5;14:2612. doi: 10.1038/s41467-023-38016-4 (PMC10163032; doi:10.1038/s41467-023-38016-4)
Supplement: Supplementary file 2 — Description of Additional Supplementary Files [file 41467_2023_38016_MOESM2_ESM.pdf]

Description of Additional Supplementary Files

Title: Supplementary Data 1.
Description: Summary statistics for single-cell data sets.

Title: Supplementary Data 2.
Description: Markers from each cluster for the postembryonic development stages as determined by Seurat.

Title: Supplementary Data 3.
Description: Stage-specific clusters in the postembryonic development scRNA-seq data as determined by Seurat.

Title: Supplementary Data 4.
Description: Cluster markers for the merged postembryonic development scRNA-seq data set as determined by Seurat and associated cluster key.

Title: Supplementary Data 5.
Description: Shared cell type expression comparison between hatchling juvenile and late juvenile populations as determined by Seurat and associated GO.

Title: Supplementary Data 6.
Description: Shared cell type expression comparison between hatchling juvenile and early adult populations as determined by Seurat and associated GO.

Title: Supplementary Data 7.
Description: Shared cell type expression comparison between hatchling juvenile and late adult populations as determined by Seurat and associated GO.

Title: Supplementary Data 8.
Description: Orthology assessment for transcription factors associated with specialized neoblast and progenitor populations.

Title: Supplementary Data 9.
Description: Cluster markers for the merged regeneration scRNA-seq data set as determined by Seurat and associated cluster key.

Title: Supplementary Data 10.
Description: Digestive cell type expression comparisons across regeneration time points as determined by Seurat and associated GO.

Title: Supplementary Data 11.
Description: Epidermal cell type expression comparisons across regeneration time points as determined by Seurat and associated GO.

Title: Supplementary Data 12.
Description: Muscle cell type expression comparisons across regeneration time points as determined by Seurat and associated GO.

Title: Supplementary Data 13.
Description: Neural cell type expression comparisons across regeneration time points as determined by Seurat and associated GO.

Title: Supplementary Data 14.
Description: Endoderm-like I cell type expression comparisons across regeneration time points as determined by Seurat and associated GO.

Title: Supplementary Data 15.
Description: Endoderm-like II/III cell type expression comparisons across regeneration time points as determined by Seurat and associated GO.

Title: Supplementary Data 16.
Description: Endodermal progenitor cell type expression comparisons across regeneration time points as determined by Seurat and associated GO.

Title: Supplementary Data 17.
Description: Epidermal progenitor cell type expression comparisons across regeneration time points as determined by Seurat and associated GO.

Title: Supplementary Data 18.
Description: Secretory cell type expression comparisons across regeneration time points as determined by Seurat and associated GO.

Title: Supplementary Data 19.
Description: Germline cell type expression comparisons across regeneration time points as determined by Seurat and associated GO.

Title: Supplementary Data 20.
Description: Neoblast cell type expression comparisons across regeneration time points as determined by Seurat and associated GO.

Title: Supplementary Data 21.
Description: Markers from the merged regeneration neoblasts subclusters as determined by Seurat.

Title: Supplementary Data 22.
Description: Neoblast subset differential gene expression 6v0hpa as determined by Seurat.

Title: Supplementary Data 23.
Description: Primer sequences for screened marker genes from the single-cell experiments.
