# Supplementary material for: Acoel single-cell atlas reveals expression dynamics and heterogeneity of adult pluripotent stem cells
Source: Nat Commun. 2023 May 5;14:2612. doi: 10.1038/s41467-023-38016-4 (PMC10163032; doi:10.1038/s41467-023-38016-4)
Supplement: Supplementary file 26 — Reporting Summary [file 41467_2023_38016_MOESM26_ESM.pdf]

nature portfolio

Corresponding author(s): Dr. Mansi Srivastava
Last updated by author(s): Mar 21, 2023

Reporting Summary

Nature Portfolio wishes to improve the reproducibility of the work that we publish. This form provides structure for consistency and transparency in reporting. For further information on Nature Portfolio policies, see our Editorial Policies and the Editorial Policy Checklist.

Statistics

For all statistical analyses, confirm that the following items are present in the figure legend, table legend, main text, or Methods section.

n/a	Confirmed	
☐	☒	The exact sample size (*n*) for each experimental group/condition, given as a discrete number and unit of measurement
☐	☒	A statement on whether measurements were taken from distinct samples or whether the same sample was measured repeatedly
☐	☒	The statistical test(s) used AND whether they are one- or two-sided<br>*Only common tests should be described solely by name; describe more complex techniques in the Methods section.*
☒	☐	A description of all covariates tested
☒	☐	A description of any assumptions or corrections, such as tests of normality and adjustment for multiple comparisons
☐	☒	A full description of the statistical parameters including central tendency (e.g. means) or other basic estimates (e.g. regression coefficient) AND variation (e.g. standard deviation) or associated estimates of uncertainty (e.g. confidence intervals)
☐	☒	For null hypothesis testing, the test statistic (e.g. *F*, *t*, *r*) with confidence intervals, effect sizes, degrees of freedom and *P* value noted<br>*Give P values as exact values whenever suitable.*
☒	☐	For Bayesian analysis, information on the choice of priors and Markov chain Monte Carlo settings
☒	☐	For hierarchical and complex designs, identification of the appropriate level for tests and full reporting of outcomes
☐	☒	Estimates of effect sizes (e.g. Cohen's *d*, Pearson's *r*), indicating how they were calculated

Our web collection on statistics for biologists contains articles on many of the points above.

Software and code

Policy information about availability of computer code

Data collection	No software used for data collection.
Data analysis	R 3.4.1, Seurat v3.1.4, Bowtie v1.1,1, RSEMv1.2.19, Python 2.7, samtools 1.3.1, pysam 0.9.1, bcl2fastq v2.18, URD v1.0.1, BLAST website

For manuscripts utilizing custom algorithms or software that are central to the research but not yet described in published literature, software must be made available to editors and reviewers. We strongly encourage code deposition in a community repository (e.g. GitHub). See the Nature Portfolio guidelines for submitting code & software for further information.

Data

Policy information about availability of data

All manuscripts must include a data availability statement. This statement should provide the following information, where applicable:

- Accession codes, unique identifiers, or web links for publicly available datasets
- A description of any restrictions on data availability
- For clinical datasets or third party data, please ensure that the statement adheres to our policy

Data generated in this study are included in this published article and supplementary files. Sequencing raw reads and processed counts matrices associated with this study will be deposited as NCBI BioProjects PRJNA888438 (postembryonic development) and PRJNA908236 (regeneration time course). Processed count matrices and R scripts are also available on Github with the zenodo DOI: 10.5281/zenodo.7700424. An online resource was generated to access and visualize the scRNA-seq, found at https://dredge.ptgolden.org/sc/?page=home.

Human research participants

Policy information about studies involving human research participants and Sex and Gender in Research.

Reporting on sex and gender	Not applicable to this study.
Population characteristics	Not applicable to this study.
Recruitment	Not applicable to this study.
Ethics oversight	Not applicable to this study.

Note that full information on the approval of the study protocol must also be provided in the manuscript.

Field-specific reporting

Please select the one below that is the best fit for your research. If you are not sure, read the appropriate sections before making your selection.

☒ Life sciences ☐ Behavioural & social sciences ☐ Ecological, evolutionary & environmental sciences

For a reference copy of the document with all sections, see nature.com/documents/nr-reporting-summary-flat.pdf

Life sciences study design

All studies must disclose on these points even when the disclosure is negative.

Sample size	Determined by the limits of the inDrops assay which states 3000 cells/library. Each experiment was done in triplicate i.e. three libraries were made per sample. For FISH experiments, experiments were done in at least triplicate with at least 5 animals per condition and repeated successfully.
Data exclusions	No data were excluded from this study.
Replication	At least 3 libraries per sample were successfully prepared for the single-cell data. For FISH experiments, experiments were done in at least triplicate with at least 5 animals per condition and repeated successfully.
Randomization	Cell clustering was performed using unbiased statistical methods and then fine-tuned based on FISH corroboration
Blinding	Blinding is not relevant because single-cell data analysis was performed using an unbiased pipeline and then corroborated by FISH.

Reporting for specific materials, systems and methods

We require information from authors about some types of materials, experimental systems and methods used in many studies. Here, indicate whether each material, system or method listed is relevant to your study. If you are not sure if a list item applies to your research, read the appropriate section before selecting a response.

Materials & experimental systems

n/a	Involved in the study	
☒	☐	Antibodies
☒	☐	Eukaryotic cell lines
☒	☐	Palaeontology and archaeology
☐	☒	Animals and other organisms
☒	☐	Clinical data
☒	☐	Dual use research of concern

Methods

n/a	Involved in the study	
☒	☐	ChIP-seq
☒	☐	Flow cytometry
☒	☐	MRI-based neuroimaging

Animals and other research organisms

Policy information about studies involving animals; ARRIVE guidelines recommended for reporting animal research, and Sex and Gender in Research

Laboratory animals	Hofstenia miamia; Ages include hatchling juveniles (14 days post lay) through late adult (>90 days post lay). Single cell data were

Laboratory animals	collected from animals from 4 different ages (as outlined in the methods). Most FISH were performed in 2-3 week old animals. Germ line FISH were performed in adult animals.
Wild animals	This study did not include wild caught animals.
Reporting on sex	The animals are hermaphrodites and sampled at random.
Field-collected samples	No samples were collected in the field for this study.
Ethics oversight	No ethical approval required because these are unregulated marine invertebrate animals.

Note that full information on the approval of the study protocol must also be provided in the manuscript.
