# Supplementary material for: Acoel single-cell atlas reveals expression dynamics and heterogeneity of adult pluripotent stem cells
Source: Nat Commun. 2023 May 5;14:2612. doi: 10.1038/s41467-023-38016-4 (PMC10163032; doi:10.1038/s41467-023-38016-4)
Supplement: Supplementary file 1 — Supplementary Information [file 41467_2023_38016_MOESM1_ESM.pdf]

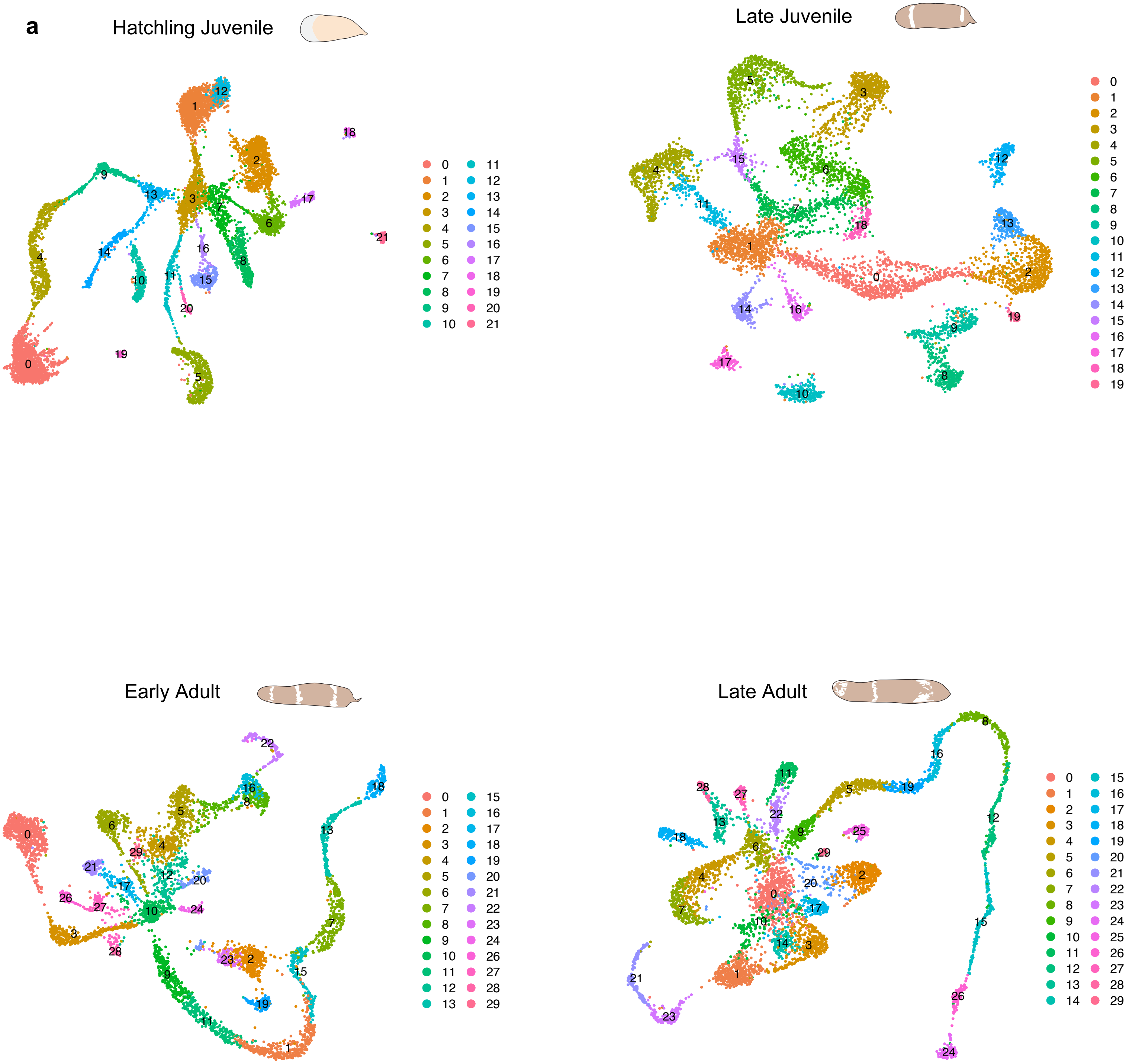
a
Hatchling Juvenile
Late Juvenile
Early Adult
Late Adult

b

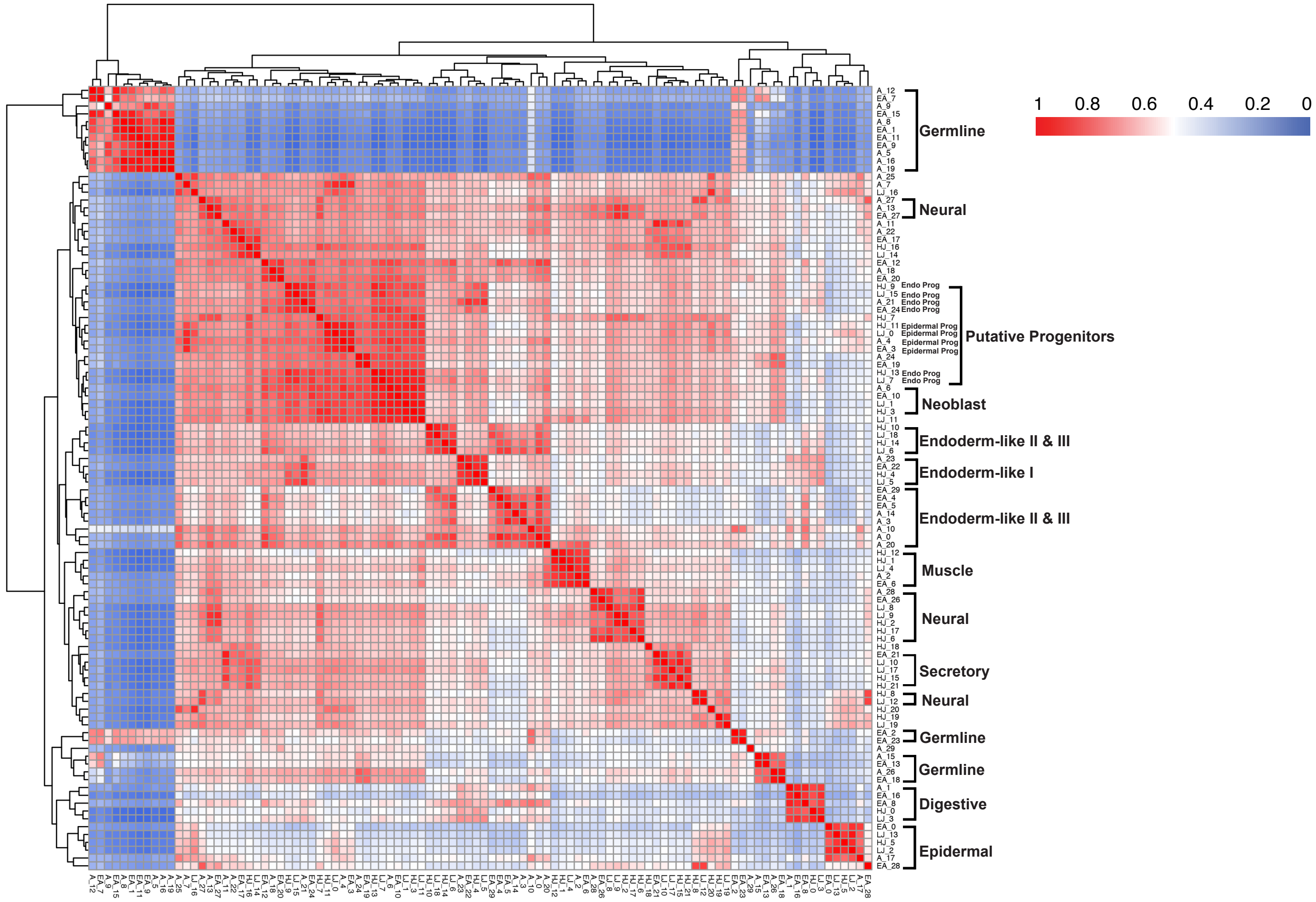
1
0.8
0.6
0.4
0.2
0
Germline
Neural
Putative Progenitors
Neoblast
Endoderm-like II & III
Endoderm-like I
Endoderm-like II & III
Muscle
Neural
Secretory
Neural
Germline
Germline
Digestive
Epidermal

c

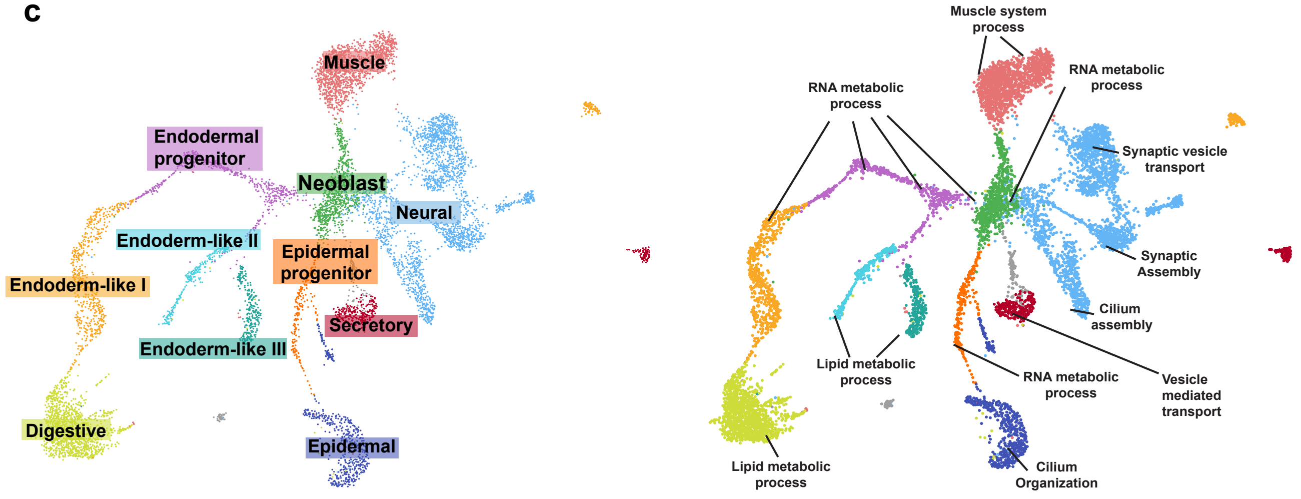
Muscle
Endodermal progenitor
Neoblast
Neural
Endoderm-like II
Epidermal progenitor
Endoderm-like I
Secretory
Endoderm-like III
Digestive
Epidermal
Muscle system process
RNA metabolic process
RNA metabolic process
Synaptic vesicle transport
Synaptic Assembly
Cilium assembly
Lipid metabolic process
RNA metabolic process
Vesicle mediated transport
Lipid metabolic process
Cilium Organization

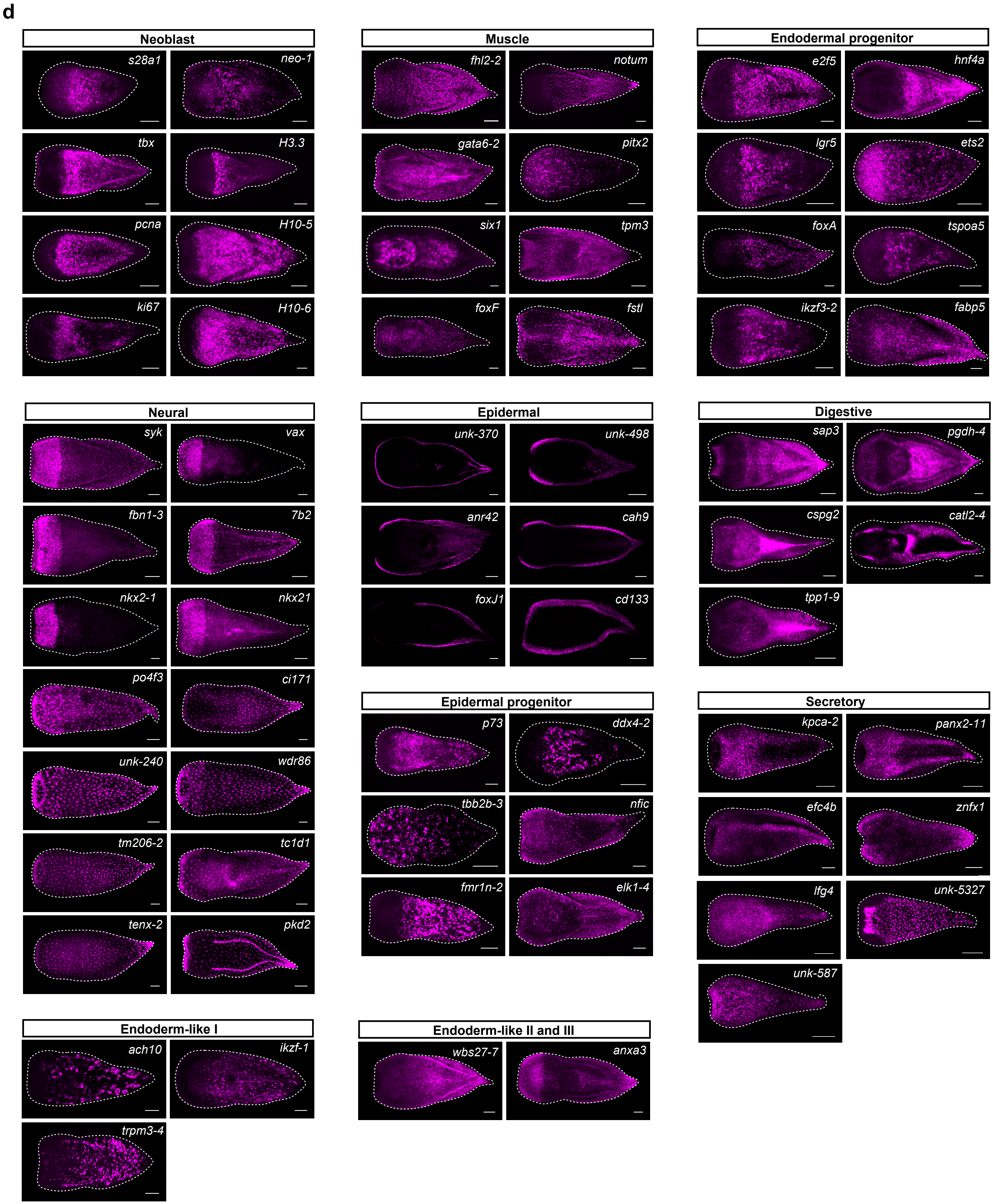
d
Neoblast
s28a1
neo-1
tbx
H3.3
pcna
H10-5
ki67
H10-6
Muscle
fhl2-2
notum
gata6-2
pitx2
six1
tpm3
foxF
fstl
Endodermal progenitor
e2f5
hnf4a
lgr5
ets2
foxA
tspoa5
ikzf3-2
fabp5
Neural
syk
vax
fbn1-3
7b2
nkx2-1
nkx21
po4f3
ci171
unk-240
wdr86
tm206-2
tc1d1
tenx-2
pkd2
Epidermal
unk-370
unk-498
anr42
cah9
foxJ1
cd133
Digestive
sap3
pgdh-4
cspg2
catl2-4
tpp1-9
Epidermal progenitor
p73
ddx4-2
tbb2b-3
nfic
fmr1n-2
elk1-4
Secretory
kpca-2
panx2-11
efc4b
znfx1
lfg4
unk-5327
unk-587
Endoderm-like I
ach10
ikzf-1
trpm3-4
Endoderm-like II and III
wbs27-7
anxa3

e

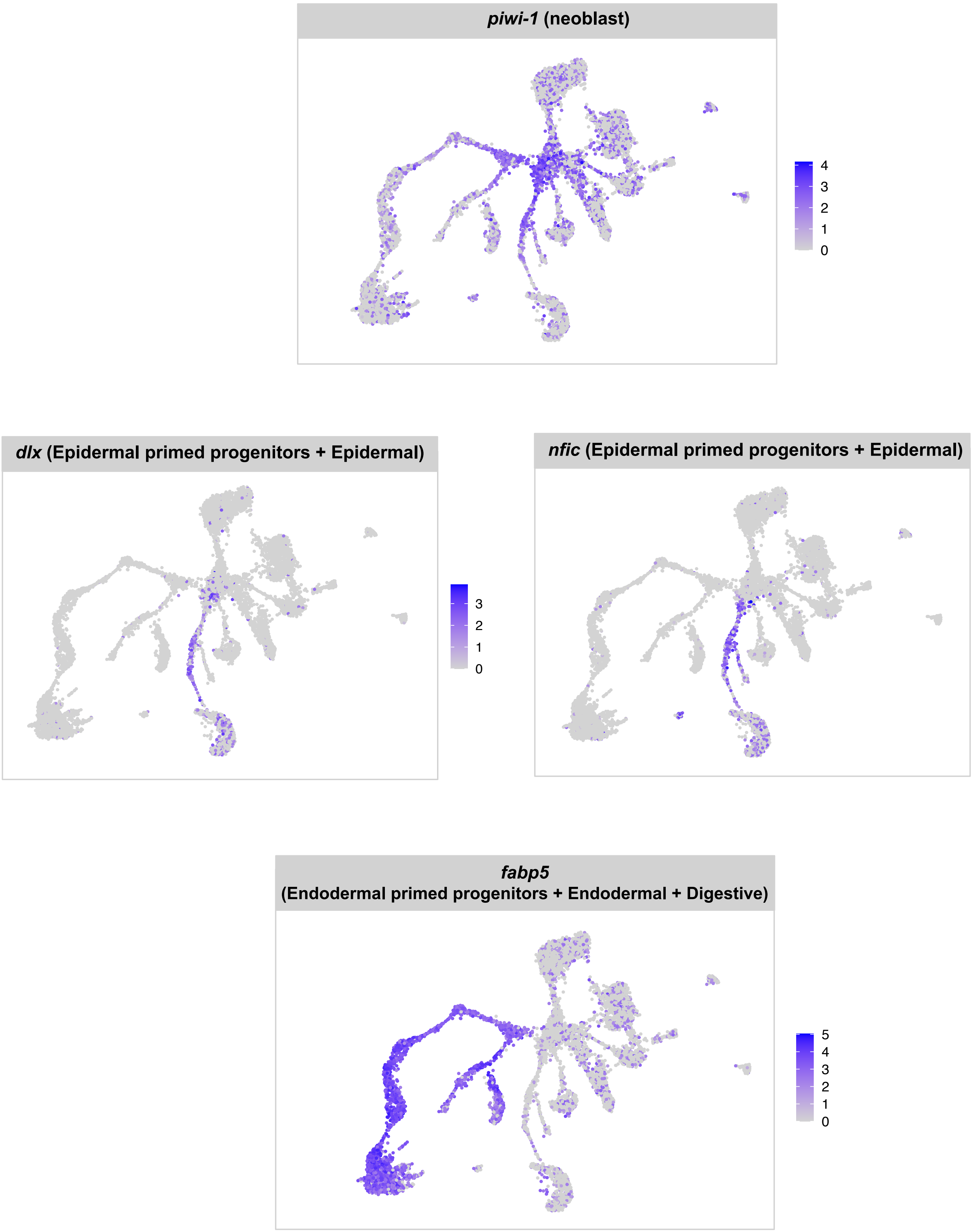
piwi-1 (neoblast)
4
3
2
1
0
dlx (Epidermal primed progenitors + Epidermal)
3
2
1
0
nfic (Epidermal primed progenitors + Epidermal)
3
2
1
0
fabp5
(Endodermal primed progenitors + Endodermal + Digestive)
5
4
3
2
1
0

Supplementary Figure 1. Identification and corroboration of shared cell types across postembryonic development (A) UMAPs representing putative cell types across postembryonic development with associated cluster numbers. Associated marker genes are in Supplementary Table 2. (B) Heatmap of Pearson correlation coefficients based on transcriptional profiles of all cell clusters from the postembryonic data sets with hierarchical clustering. HJ= hatchling juvenile, LJ= late juvenile, EA= early adult, A= Adult. (C) Left: Hatchling juvenile UMAP with putative cluster labels. Right: Gene ontology (GO) terms associated with each hatchling juvenile cluster based on highly expressed genes found within each cluster. (D) FISH of genes associated with each cluster, with markers from the same cell clusters showing similar gene expression patterns. Scale bars 100 µm. (E) UMAP showing *piwi-1* gene expression with enrichment in the central neoblast cluster as well as the putative epidermal and endodermal lineage-primed progenitors (top). UMAPs of Epidermal lineage-primed progenitor and differentiated populations depicted with *dlx* and *nfic* expression (middle). UMAP of endodermal lineage-primed progenitor and differentiated endodermal + digestive cells depicted with *fabp5* expression (bottom).

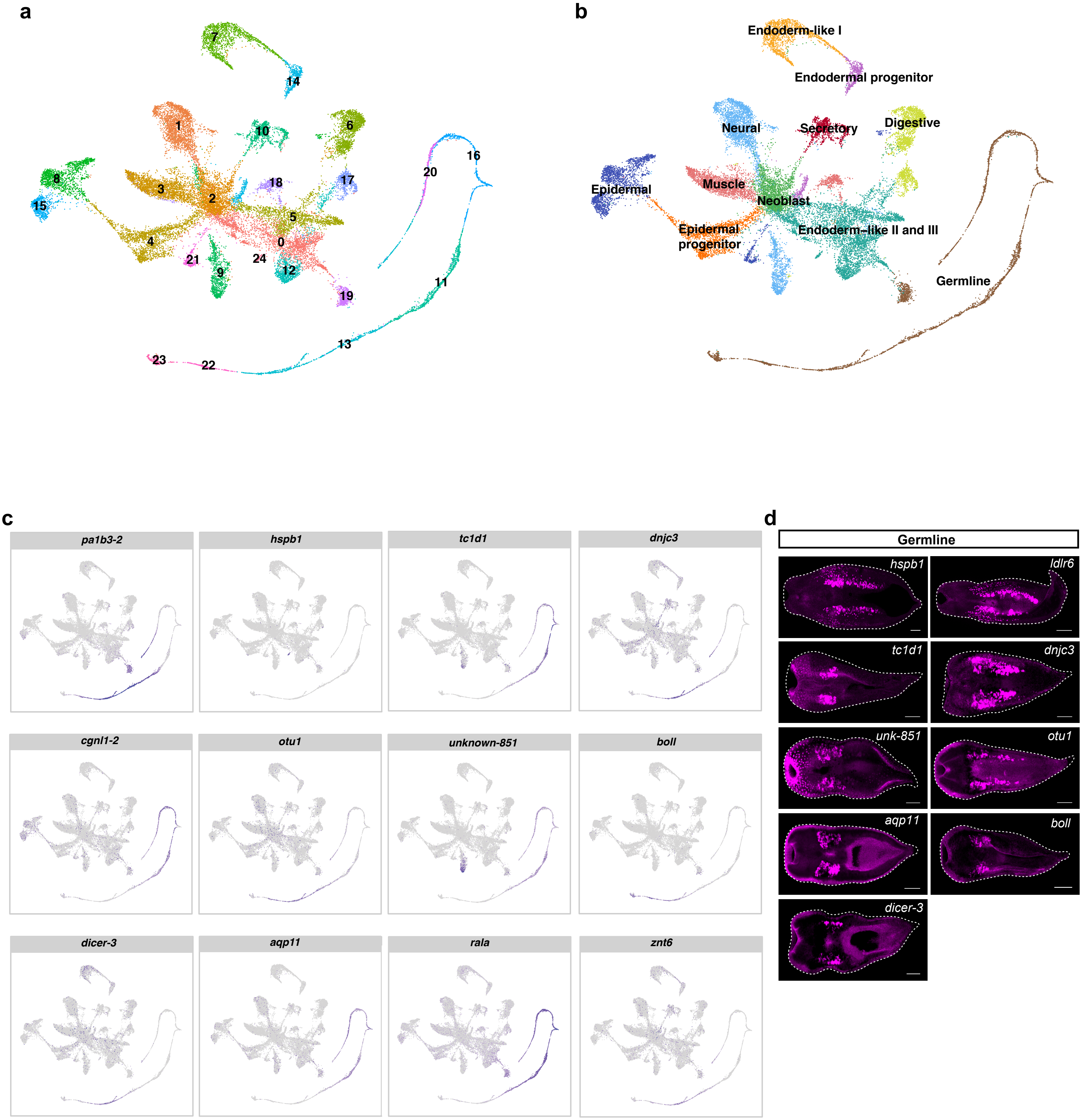

a
7
14
1
10
6
8
3
2
18
17
16
20
15
5
4
0
21
24
12
9
19
11
13
23
22
b
Endoderm-like I
Endodermal progenitor
Neural
Secretory
Digestive
Epidermal
Muscle
Neoblast
Epidermal progenitor
Endoderm–like II and III
Germline
c
pa1b3-2
hspb1
tc1d1
dnjc3
cgnl1-2
otu1
unknown-851
boll
dicer-3
aqp11
rala
znt6
d
Germline
hspb1
ldlr6
tc1d1
dnjc3
unk-851
otu1
aqp11
boll
dicer-3

Supplementary Figure 2. Merged postembryonic development UMAP recapitulates major shared cell types and highlights the presence of germline cells in adults (A) Merged postembryonic development UMAP with associated cluster numbers. (B) UMAP showing the 11 major shared cell types as well as the adult specific germline population. (C) Projections of putative germline markers in the merged postembryonic scRNA-seq data set. (D) FISH corroboration of germline markers in adult animals. Scale bars 200 μm. Germline clusters are annotated based on reconciliation of gene expression patterns in FISH experiments with the projection of gene expression onto the UMAP. Marker genes did not enable disambiguation of male versus female germline clusters.

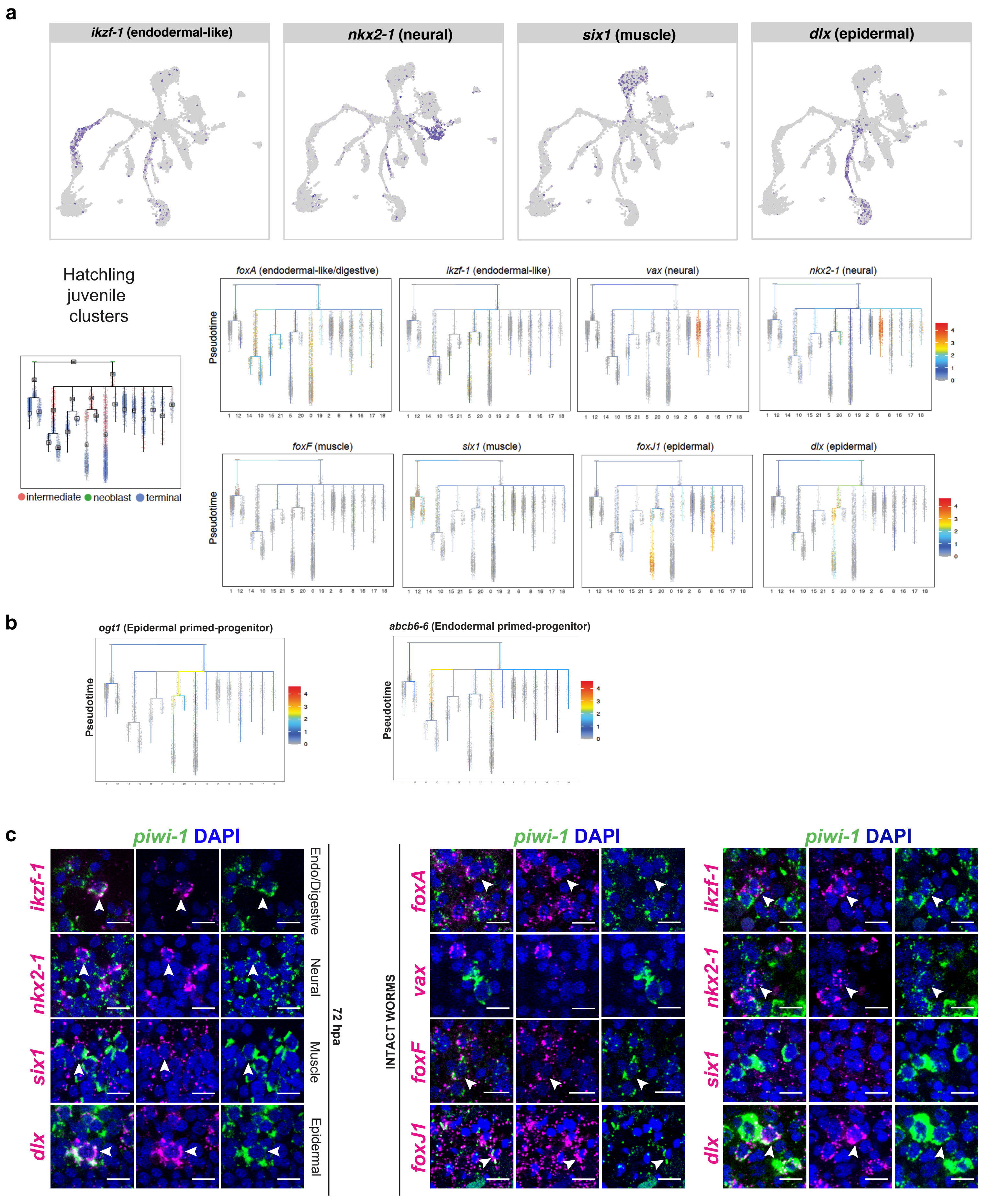

a
ikzf-1 (endodermal-like)
nkx2-1 (neural)
six1 (muscle)
dlx (epidermal)
Hatchling juvenile clusters
intermediate
neoblast
terminal
foxA (endodermal-like/digestive)
ikzf-1 (endodermal-like)
vax (neural)
nkx2-1 (neural)
foxF (muscle)
six1 (muscle)
foxJ1 (epidermal)
dlx (epidermal)
Pseudotime
b
ogt1 (Epidermal primed-progenitor)
abcb6-6 (Endodermal primed-progenitor)
c
piwi-1 DAPI
ikzf-1
nkx2-1
six1
dlx
Endo/Digestive
Neural
Muscle
Epidermal
72 hpa
INTACT WORMS
foxA
vax
foxF
foxJ1

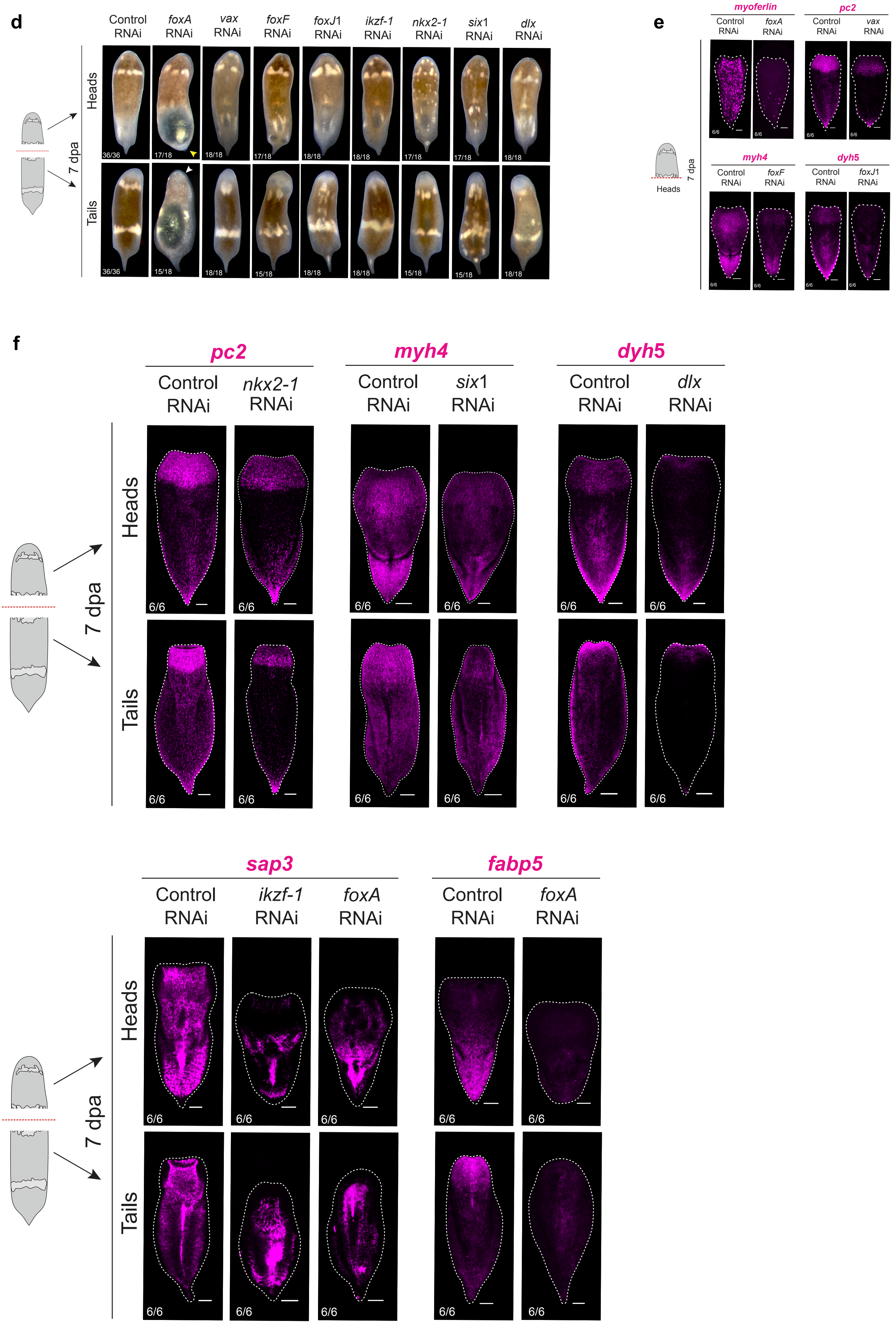

d
Control RNAi
foxA RNAi
vax RNAi
foxF RNAi
foxJ1 RNAi
ikzf-1 RNAi
nkx2-1 RNAi
six1 RNAi
dlx RNAi
Heads
Tails
7 dpa
36/36
17/18
18/18
17/18
18/18
18/18
17/18
17/18
18/18
36/36
15/18
18/18
15/18
18/18
18/18
15/18
15/18
18/18
e
myoferlin
pc2
myh4
dyh5
Control RNAi
foxA RNAi
vax RNAi
foxF RNAi
foxJ1 RNAi
Heads
7 dpa
6/6
f
pc2
myh4
dyh5
Control RNAi
nkx2-1 RNAi
six1 RNAi
dlx RNAi
Heads
Tails
7 dpa
6/6
sap3
fabp5
ikzf-1 RNAi
foxA RNAi

g

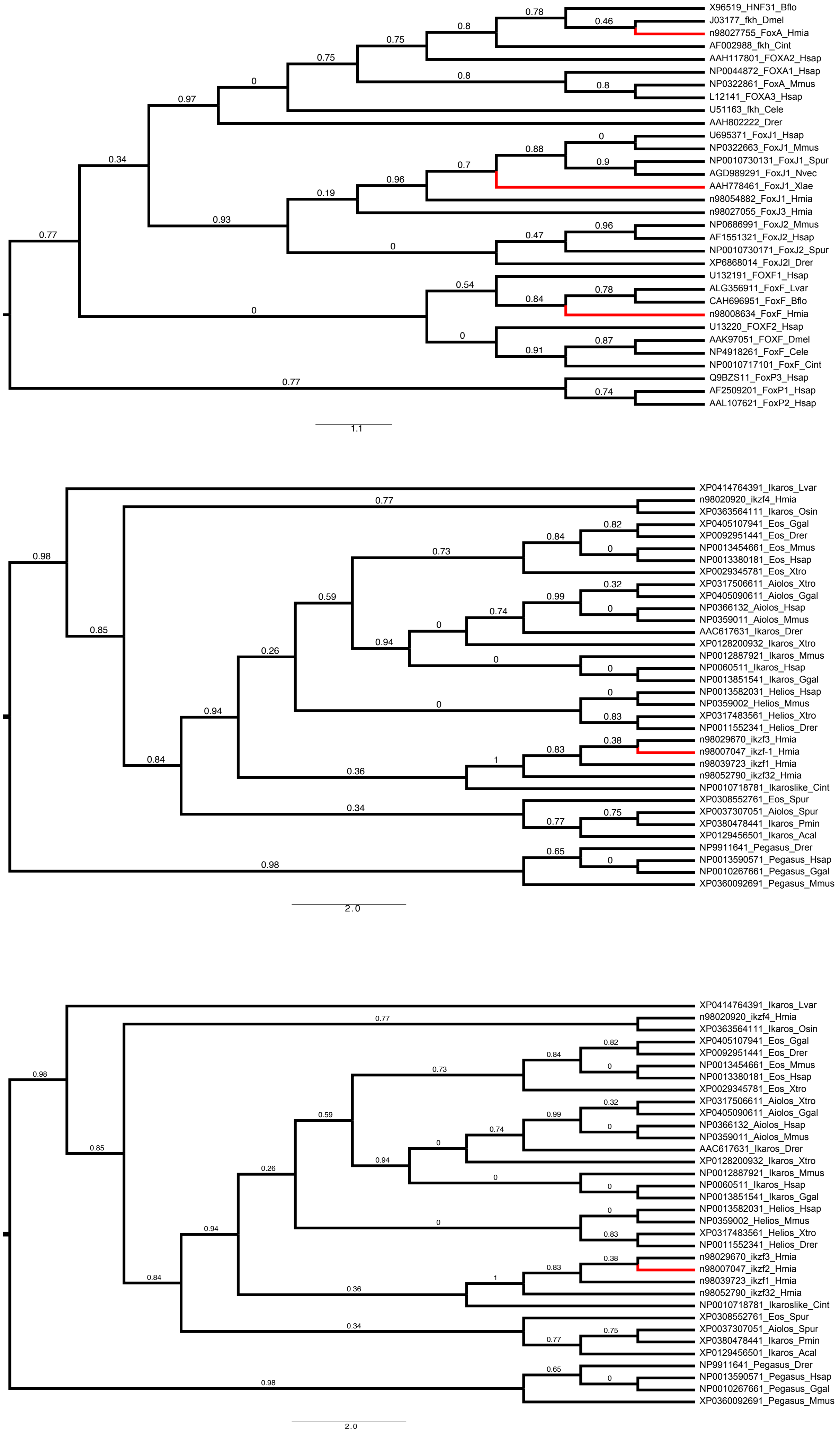
X96519_HNF31_Bflo
J03177_fkh_Dmel
n98027755_FoxA_Hmia
AF002988_fkh_Cint
AAH117801_FOXA2_Hsap
NP0044872_FOXA1_Hsap
NP0322861_FoxA_Mmus
L12141_FOXA3_Hsap
U51163_fkh_Cele
AAH802222_Drer
U695371_FoxJ1_Hsap
NP0322663_FoxJ1_Mmus
NP0010730131_FoxJ1_Spur
AGD989291_FoxJ1_Nvec
AAH778461_FoxJ1_Xlae
n98054882_FoxJ1_Hmia
n98027055_FoxJ3_Hmia
NP0686991_FoxJ2_Mmus
AF1551321_FoxJ2_Hsap
NP0010730171_FoxJ2_Spur
XP6868014_FoxJ2l_Drer
U132191_FOXF1_Hsap
ALG356911_FoxF_Lvar
CAH696951_FoxF_Bflo
n98008634_FoxF_Hmia
U13220_FOXF2_Hsap
AAK97051_FOXF_Dmel
NP4918261_FoxF_Cele
NP0010717101_FoxF_Cint
Q9BZS11_FoxP3_Hsap
AF2509201_FoxP1_Hsap
AAL107621_FoxP2_Hsap
1.1
XP0414764391_Ikaros_Lvar
n98020920_ikzf4_Hmia
XP0363564111_Ikaros_Osin
XP0405107941_Eos_Ggal
XP0092951441_Eos_Drer
NP0013454661_Eos_Mmus
NP0013380181_Eos_Hsap
XP0029345781_Eos_Xtro
XP0317506611_Aiolos_Xtro
XP0405090611_Aiolos_Ggal
NP0366132_Aiolos_Hsap
NP0359011_Aiolos_Mmus
AAC617631_Ikaros_Drer
XP0128200932_Ikaros_Xtro
NP0012887921_Ikaros_Mmus
NP0060511_Ikaros_Hsap
NP0013851541_Ikaros_Ggal
NP0013582031_Helios_Hsap
NP0359002_Helios_Mmus
XP0317483561_Helios_Xtro
NP0011552341_Helios_Drer
n98029670_ikzf3_Hmia
n98007047_ikzf-1_Hmia
n98039723_ikzf1_Hmia
n98052790_ikzf32_Hmia
NP0010718781_Ikaroslike_Cint
XP0308552761_Eos_Spur
XP0037307051_Aiolos_Spur
XP0380478441_Ikaros_Pmin
XP0129456501_Ikaros_Acal
NP9911641_Pegasus_Drer
NP0013590571_Pegasus_Hsap
NP0010267661_Pegasus_Ggal
XP0360092691_Pegasus_Mmus
2.0
XP0414764391_Ikaros_Lvar
n98020920_ikzf4_Hmia
XP0363564111_Ikaros_Osin
XP0405107941_Eos_Ggal
XP0092951441_Eos_Drer
NP0013454661_Eos_Mmus
NP0013380181_Eos_Hsap
XP0029345781_Eos_Xtro
XP0317506611_Aiolos_Xtro
XP0405090611_Aiolos_Ggal
NP0366132_Aiolos_Hsap
NP0359011_Aiolos_Mmus
AAC617631_Ikaros_Drer
XP0128200932_Ikaros_Xtro
NP0012887921_Ikaros_Mmus
NP0060511_Ikaros_Hsap
NP0013851541_Ikaros_Ggal
NP0013582031_Helios_Hsap
NP0359002_Helios_Mmus
XP0317483561_Helios_Xtro
NP0011552341_Helios_Drer
n98029670_ikzf3_Hmia
n98007047_ikzf2_Hmia
n98039723_ikzf1_Hmia
n98052790_ikzf32_Hmia
NP0010718781_Ikaroslike_Cint
XP0308552761_Eos_Spur
XP0037307051_Aiolos_Spur
XP0380478441_Ikaros_Pmin
XP0129456501_Ikaros_Acal
NP9911641_Pegasus_Drer
NP0013590571_Pegasus_Hsap
NP0010267661_Pegasus_Ggal
XP0360092691_Pegasus_Mmus
2.0

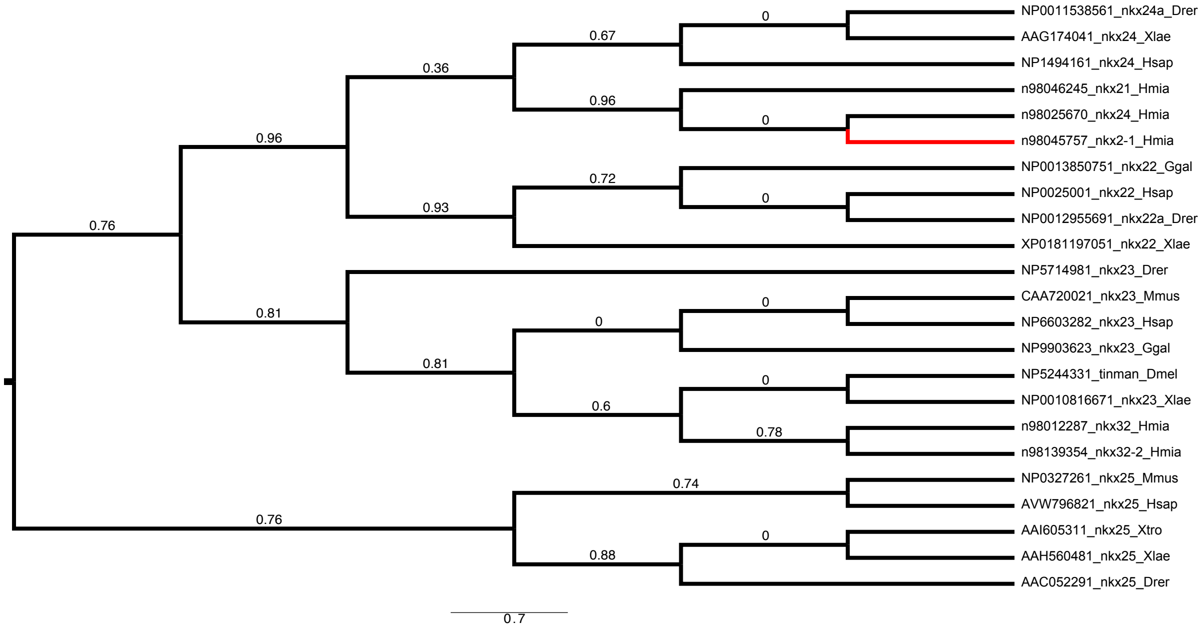
NP0011538561_nkx24a_Drer
AAG174041_nkx24_Xlae
NP1494161_nkx24_Hsap
n98046245_nkx21_Hmia
n98025670_nkx24_Hmia
n98045757_nkx2-1_Hmia
NP0013850751_nkx22_Ggal
NP0025001_nkx22_Hsap
NP0012955691_nkx22a_Drer
XP0181197051_nkx22_Xlae
NP5714981_nkx23_Drer
CAA720021_nkx23_Mmus
NP6603282_nkx23_Hsap
NP9903623_nkx23_Ggal
NP5244331_tinman_Dmel
NP0010816671_nkx23_Xlae
n98012287_nkx32_Hmia
n98139354_nkx32-2_Hmia
NP0327261_nkx25_Mmus
AVW796821_nkx25_Hsap
AAI605311_nkx25_Xtro
AAH560481_nkx25_Xlae
AAC052291_nkx25_Drer
0.7

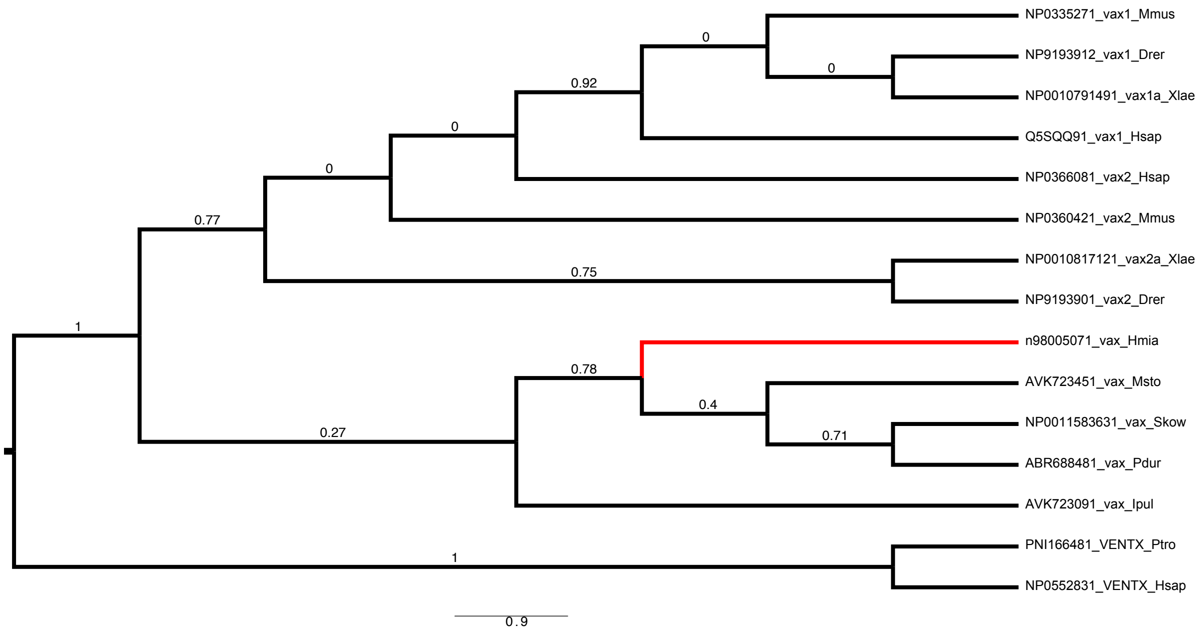
NP0335271_vax1_Mmus
NP9193912_vax1_Drer
NP0010791491_vax1a_Xlae
Q5SQQ91_vax1_Hsap
NP0366081_vax2_Hsap
NP0360421_vax2_Mmus
NP0010817121_vax2a_Xlae
NP9193901_vax2_Drer
n98005071_vax_Hmia
AVK723451_vax_Msto
NP0011583631_vax_Skow
ABR688481_vax_Pdur
AVK723091_vax_Ipul
PNI166481_VENTX_Ptro
NP0552831_VENTX_Hsap
0.9

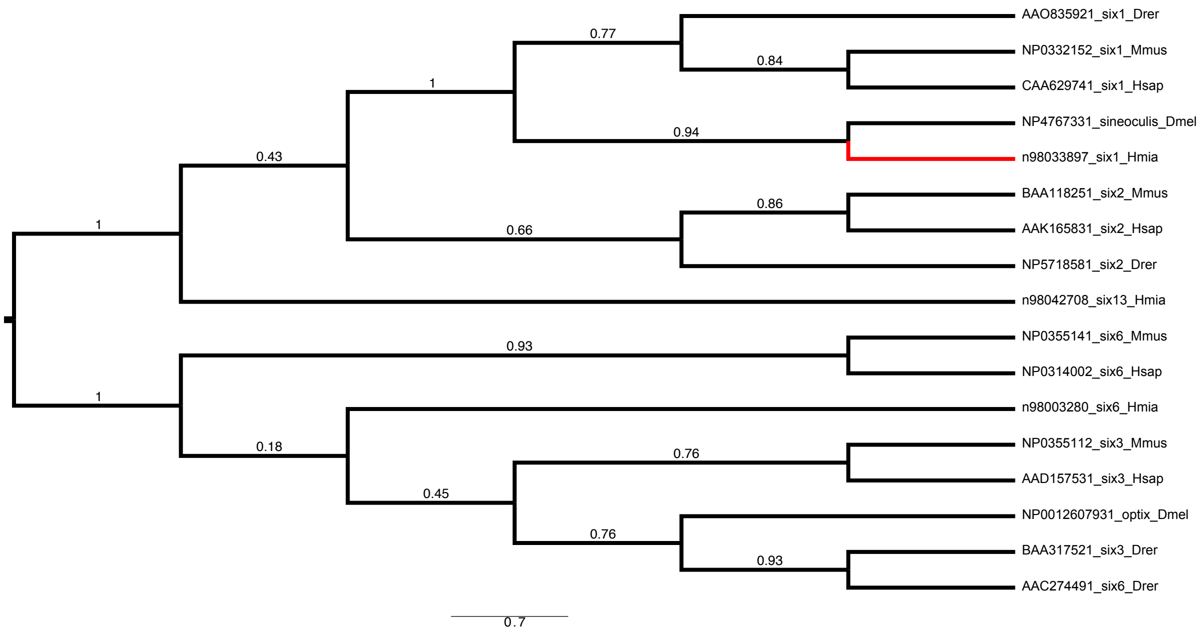
AAO835921_six1_Drer
NP0332152_six1_Mmus
CAA629741_six1_Hsap
NP4767331_sineoculis_Dmel
n98033897_six1_Hmia
BAA118251_six2_Mmus
AAK165831_six2_Hsap
NP5718581_six2_Drer
n98042708_six13_Hmia
NP0355141_six6_Mmus
NP0314002_six6_Hsap
n98003280_six6_Hmia
NP0355112_six3_Mmus
AAD157531_six3_Hsap
NP0012607931_optix_Dmel
BAA317521_six3_Drer
AAC274491_six6_Drer
0.7

Supplementary Figure 3. Additional transcription factors expressed in putative specialized neoblast populations with gene expression studies (A) UMAP projection of transcription factors expressed in putative differentiation trajectories from URD analysis along with URD trajectories. URD schematic on the left depicts intermediate (pink), neoblast (green), and terminal (blue) cell assignments for trajectory inference of hatchling juvenile data. All transcription factors chosen as candidates were found to be expressed in their corresponding differentiated cell types and/or putative specialized neoblast populations. (B) URD trajectories of putative lineage-primed progenitors for the epidermal and endodermal lineages were placed in close relationship to differentiated epidermal and digestive cells. (C) Double FISH of the candidate transcription factors with the stem cell marker *piwi-1* assessing co-expression (denoted by white arrowheads) during regeneration at 72hpa for putative endodermal-like/digestive, neural, muscle, and epidermal specialized neoblasts (left) and in intact animals (right). Scale bars 10 μm. (D) External morphological assessment of regenerating fragments at 7dpa shows animals with blastemas and regenerated heads and tails in every RNAi condition except for *foxA* RNAi, where animals failed to regenerate head blastemas (denoted by white arrowhead) and pointed tails (denoted by yellow arrowhead). (E) RNAi of putative specialized neoblast transcription factor leads to loss of differentiated cell type expression during regeneration. Regenerating head fragments are shown 7dpa corresponding to the tail fragments in Fig. 3c. Scale bars 100 μm. (F) Additional RNAi of putative specialized neoblast transcription factor leads to loss or reduction of differentiated cell type expression during regeneration. Regenerating head and tail fragments are shown 7dpa with cell-type gene expression denoted in magenta. Scale bars 100 μm. (G) Phylogenetic trees for transcription factors associated with putative specialized neoblasts. Schematics of regenerating *Hofstenia* fragments in (D-F) reprinted from Cell Reports, Volume 32, Issue 9, Ramirez, A.N., Loubet-Senear, K., & Srivastava, M., A Regulatory Program for Initiation of Wnt Signaling during Posterior Regeneration, 108098, Copyright (2020), with permission from Elsevier.

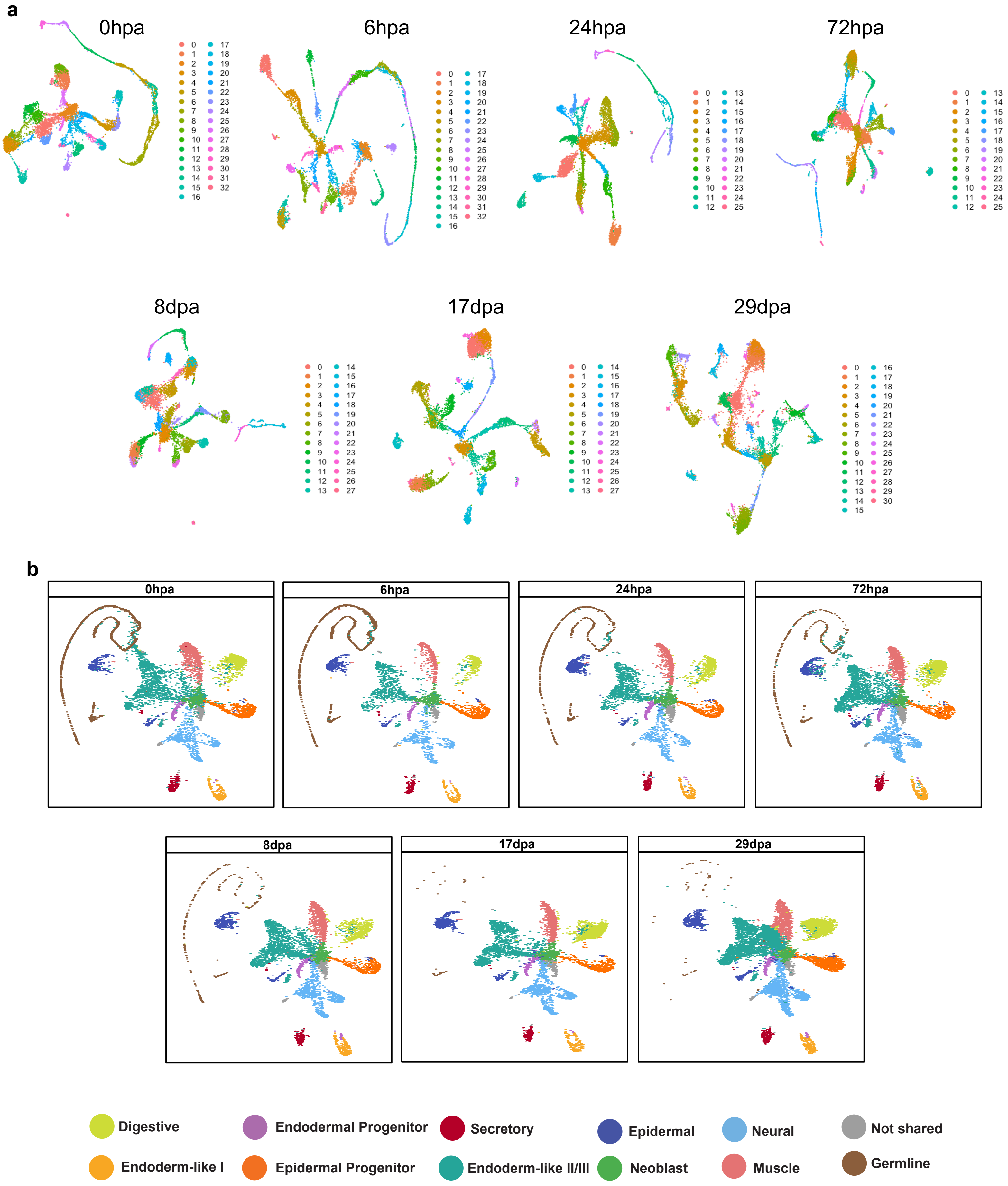
a
0hpa
6hpa
24hpa
72hpa
8dpa
17dpa
29dpa
b
0hpa
6hpa
24hpa
72hpa
8dpa
17dpa
29dpa
Digestive
Endodermal Progenitor
Secretory
Epidermal
Neural
Not shared
Endoderm-like I
Epidermal Progenitor
Endoderm-like II/III
Neoblast
Muscle
Germline

c

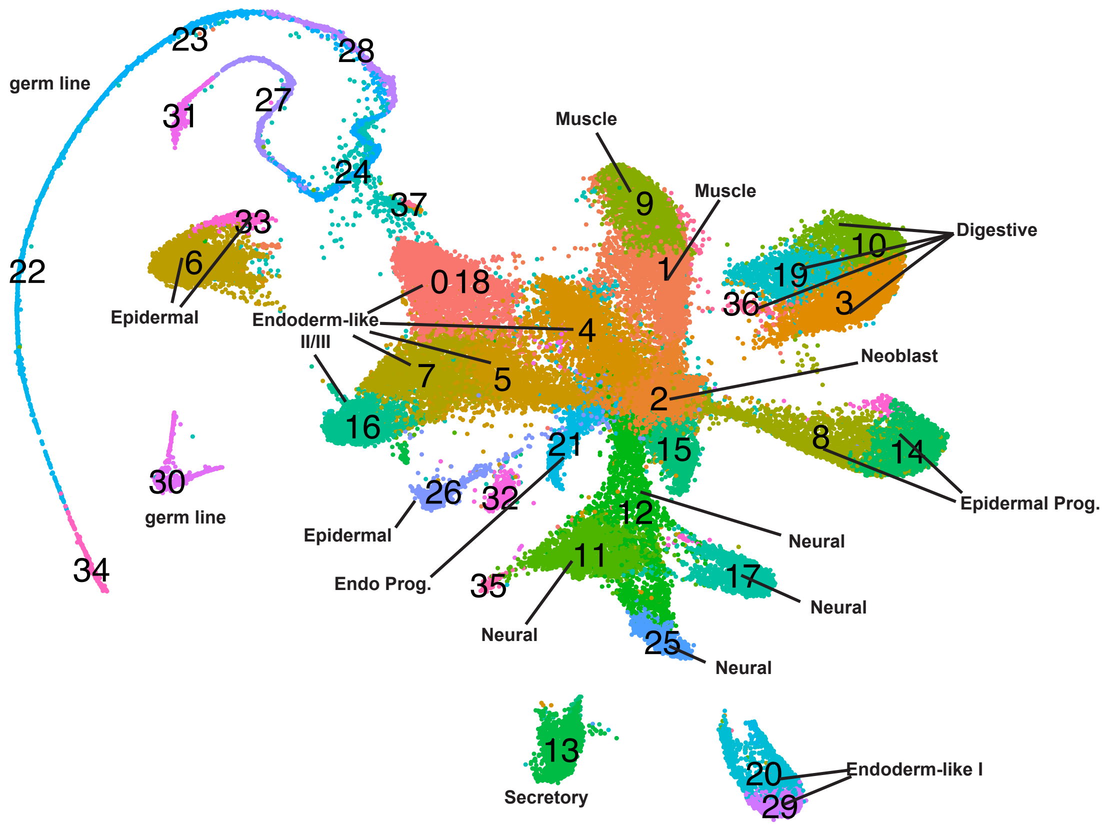
germ line
23
28
31
27
24
37
Muscle
9
Muscle
1
33
6
22
Epidermal
Endoderm-like
II/III
0
18
4
7
5
16
2
Digestive
10
19
36
3
Neoblast
8
14
Epidermal Prog.
21
15
30
germ line
26
32
Epidermal
12
Neural
34
Endo Prog.
35
11
Neural
17
Neural
25
Neural
13
Secretory
20
29
Endoderm-like I

d

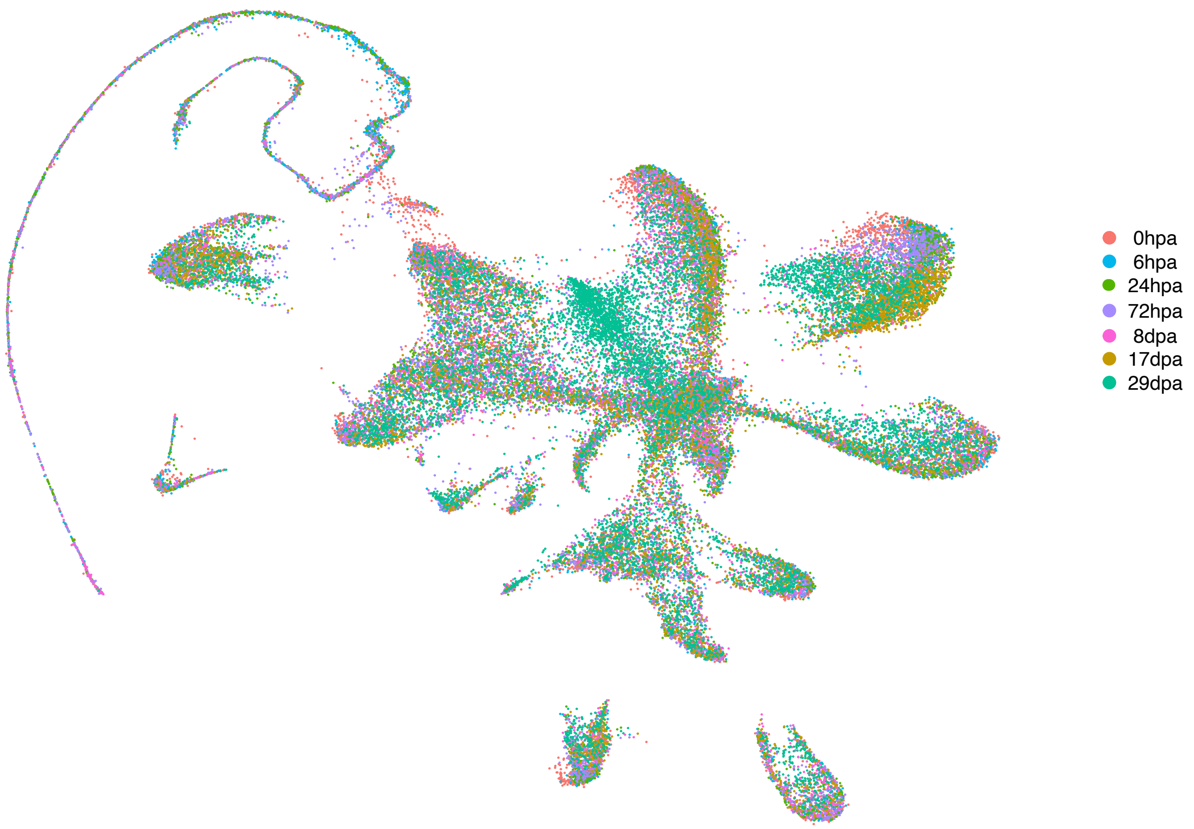
0hpa
6hpa
24hpa
72hpa
8dpa
17dpa
29dpa

e

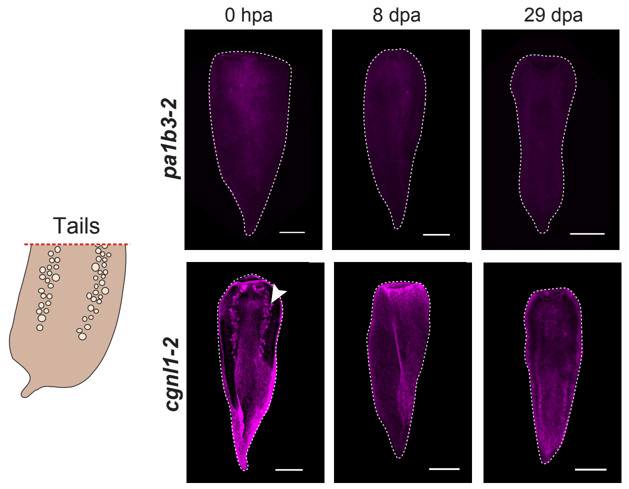
0 hpa
8 dpa
29 dpa
pa1b3-2
cgnl1-2
Tails

f

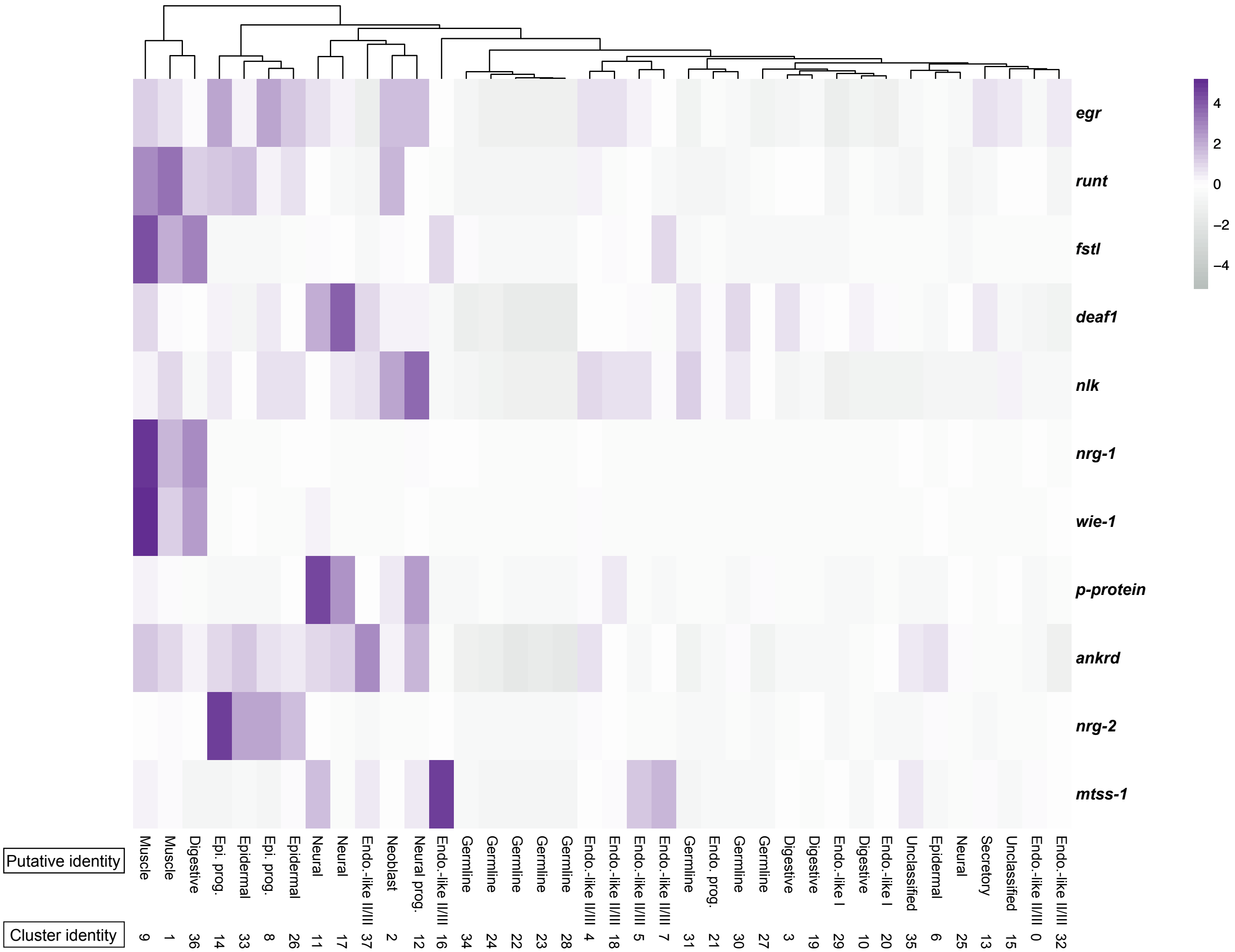
egr
runt
fstl
deaf1
nlk
nrg-1
wie-1
p-protein
ankrd
nrg-2
mtss-1
4
2
0
−2
−4
Putative identity
Cluster identity
Muscle 9
Muscle 1
Digestive 36
Epi. prog. 14
Epidermal 33
Epi. prog. 8
Epidermal 26
Neural 11
Neural 17
Endo.-like II/III 37
Neoblast 2
Neural prog. 12
Endo.-like II/III 16
Germline 34
Germline 24
Germline 22
Germline 23
Germline 28
Endo.-like II/III 4
Endo.-like II/III 18
Endo.-like II/III 5
Endo.-like II/III 7
Germline 31
Endo. prog. 21
Germline 30
Germline 27
Digestive 3
Digestive 19
Endo.-like I 29
Digestive 10
Endo.-like I 20
Unclassified 35
Epidermal 6
Neural 25
Secretory 13
Unclassified 15
Endo.-like II/III 0
Endo.-like II/III 32

Supplementary Figure 4. Merged regeneration UMAP of scRNA-seq and cluster-specific responses to the Egr-GRN (A) UMAPs representing putative cell types across regeneration with associated cluster numbers. (B) UMAP of merged regeneration scRNA-seq data colored by shared cell types over the regeneration time course sampled showing changes in the germline population over time. (C) UMAP of merged regeneration scRNA-seq data. Associated marker genes are in Supplementary Table 9. (D) UMAP of merged regeneration scRNA-seq data composed of cells from each of the regeneration time points, with no regeneration-specific cluster clearly identifiable. (E) Left: Schematic of ventral view of adult tail fragment with putative reproductive structures in white. Right: FISH of germline markers *pa1b3-2* (top) and *cgnl1-2* (bottom) in regenerating adult tail fragments over the course of 29 days (expression denoted by white arrowhead; background expression seen in *cgnl1-2* at 8dpa and 29dpa). Scale bars 200 µm. (F) Cluster-specific expression of Egr-GRN. Cells from the merged regeneration data were used to generate a heatmap depicting average expression, with putative identity labeled based on similarity to the shared cell types from the postembryonic data and cluster number from the UMAP in Fig. S4c.

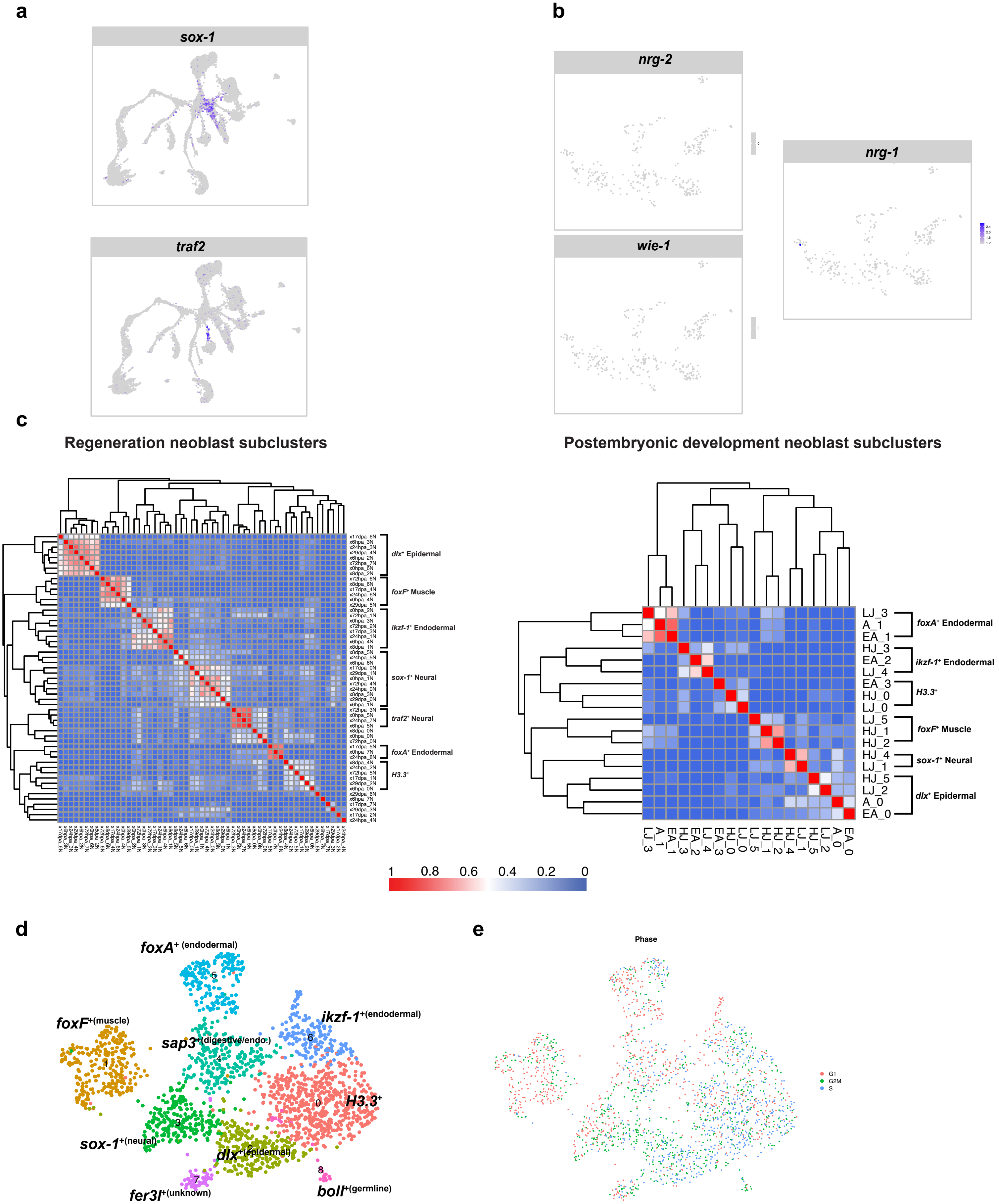
a
sox-1
traf2
b
nrg-2
wie-1
nrg-1
c
Regeneration neoblast subclusters
Postembryonic development neoblast subclusters
dlx+ Epidermal
foxF+ Muscle
ikzf-1+ Endodermal
sox-1+ Neural
traf2+ Neural
foxA+ Endodermal
H3.3+
1
0.8
0.6
0.4
0.2
0
d
foxA+ (endodermal)
foxF+(muscle)
sap3+(digestive/endo.)
ikzf-1+(endodermal)
H3.3+
sox-1+(neural)
dlx+(epidermal)
fer3l+(unknown)
boll+(germline)
e
Phase
G1
G2M
S

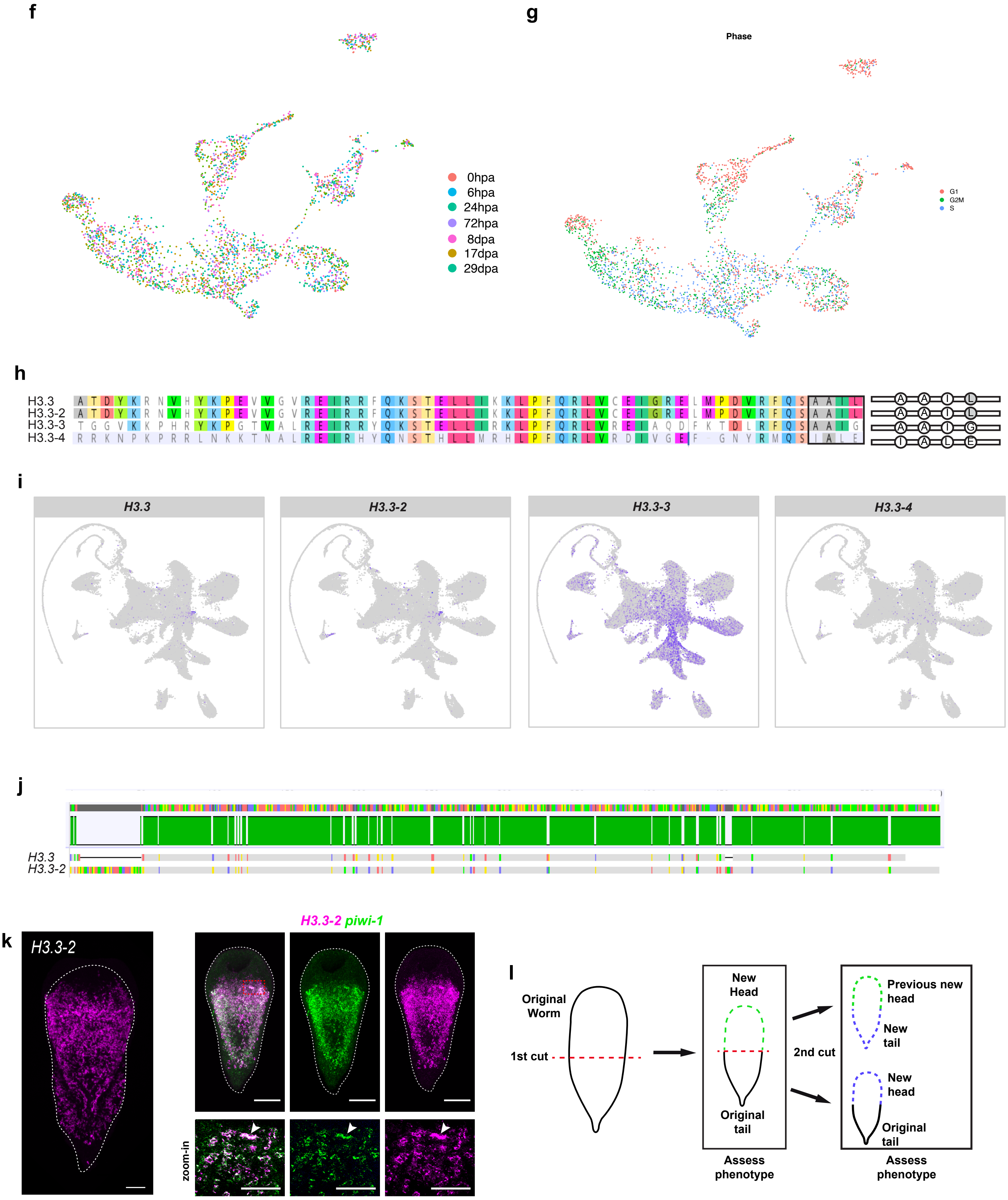
f
0hpa
6hpa
24hpa
72hpa
8dpa
17dpa
29dpa
g
Phase
G1
G2M
S
h
H3.3
H3.3-2
H3.3-3
H3.3-4
i
H3.3
H3.3-2
H3.3-3
H3.3-4
j
H3.3
H3.3-2
k
H3.3-2
H3.3-2 piwi-1
zoom-in
l
Original Worm
1st cut
New Head
Original tail
2nd cut
Previous new head
New tail
New head
Original tail
Assess phenotype
Assess phenotype

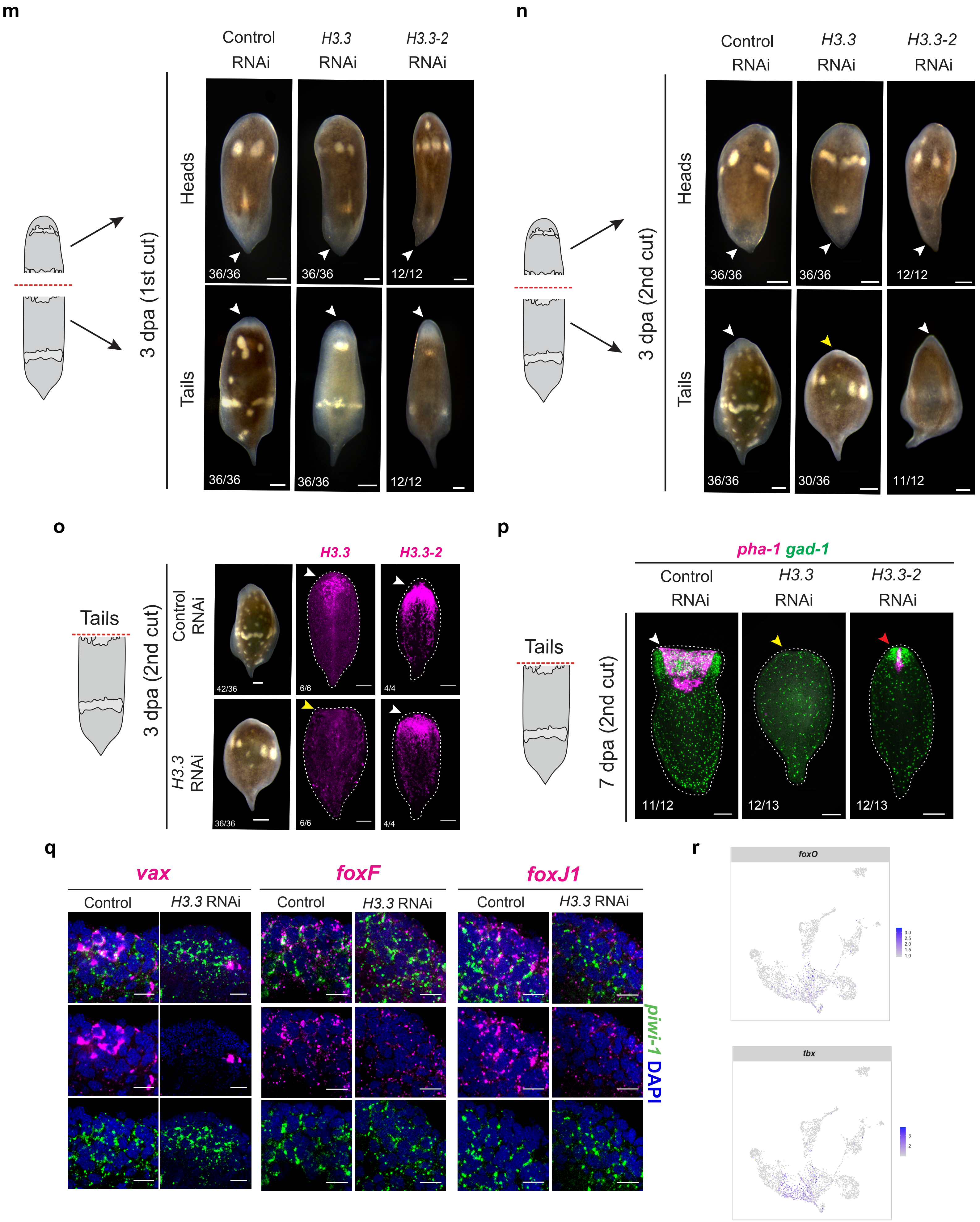
m
Control RNAi
H3.3 RNAi
H3.3-2 RNAi
3 dpa (1st cut)
Heads
Tails
36/36
36/36
12/12
36/36
36/36
12/12
n
Control RNAi
H3.3 RNAi
H3.3-2 RNAi
3 dpa (2nd cut)
Heads
Tails
36/36
36/36
12/12
36/36
30/36
11/12
o
Tails
3 dpa (2nd cut)
H3.3
H3.3-2
Control RNAi
H3.3 RNAi
42/36
6/6
4/4
36/36
6/6
4/4
p
pha-1 gad-1
Control RNAi
H3.3 RNAi
H3.3-2 RNAi
Tails
7 dpa (2nd cut)
11/12
12/13
12/13
q
vax
foxF
foxJ1
Control
H3.3 RNAi
Control
H3.3 RNAi
Control
H3.3 RNAi
piwi-1 DAPI
r
foxO
tbx

Supplementary Figure 5. Specialized neoblasts in postembryonic development and regeneration (A) Projection of *sox-1* and *traf2* genes in the hatchling juvenile UMAP supports their putative neural identity. (B) Egr-GRN members *nrg-2*, *nrg-1,* and *wie-1* are not expressed in neoblasts at 6hpa in Fig. 5b so they were not included in the heatmap. (C) Heatmaps of Pearson correlation coefficients based on transcriptional profiles of all neoblast subpopulations from the regeneration (left) and postembryonic (right) neoblasts. (D) UMAP of merged postembryonic development neoblast subpopulations with differentially expressed marker gene overlaid. (E) UMAP of merged postembryonic development neoblasts showing cell cycle phase. (F) UMAP of merged regeneration neoblast subpopulations showing that clusters are composed of cells from each of the regeneration time points, including the *H3.3*$^{+}$ population. (G) UMAP of merged regeneration neoblasts showing cell cycle phase. (H) Left: Alignment of H3.3 variant protein sequences from *Hofstenia miamia*. Previous work[100] highlights a major diagnostic character, the core histone fold. Right: A schematic of the amino acid residues at this core histone fold showing that *Hofstenia* does have a H3.3 variant with the AAIG residues and, in addition, H3.3 variants (named *H3.3* and *H3.3-2*) with a G->L substitution. (I) UMAP of merged regeneration scRNA-seq data showing *H3.3* variant gene expression with *H3.3* and *H3.3-2* enriched and expressed in the neoblast population. (J) Nucleotide alignment of *H3.3* and *H3.3-2* with distinct sequence differences. (K) Left: FISH reveals that *H3.3-2* (magenta) is expressed more broadly and lacks expression in large, midbody cells relative to *H3.3*. Scale bar 100 μm. Right: Double FISH of *H3.3-2* (magenta) and *piwi-1* (green) corroborate broad expression of *H3.3-2* and shows high level co-expression (denoted by white arrowhead). Zoom-in region denoted by red dotted square. Scale bars 100 μm for 10x magnification and 50 μm for zoom-in. (L) Diagram depicting the schema for single and double amputations used during RNAi. (M) RNAi of putative unspecialized neoblast subset markers, *H3.3* and *H3.3-2*, leads to no major impact on the visible morphology of the regenerating head and tail fragments after the first amputation. There is proper blastema formation in all regenerating fragments (denoted by white arrowheads). Regenerating head and tail fragments are shown 3 days post amputation (3dpa). Scale bars 100 μm. (N) RNAi of putative unspecialized neoblast subset markers, *H3.3* and *H3.3-2*, leads to proper blastema formation (denoted by white arrowhead) in all *H3.3-2* fragments and head fragments from *H3.3*; however, there is a lack of blastema formation (denoted by yellow arrowhead) found in *H3.3* tail fragments after a second amputation. Regenerating head and tail fragments are shown 3 days post amputation (3dpa). Scale bars 100 μm. (O) RNAi of putative unspecialized neoblast subset marker, *H3.3*, corroborated by FISH with expression in the anterior region of the control fragment (denoted by white

arrowhead) and lack of expression in the anterior region of the *H3.3* RNAi tail fragment (denoted by yellow arrowhead). *H3.3-2* expression does not seem impacted in the *H3.3* RNAi condition (denoted by white arrowheads). Regenerating tail fragments are shown 3 days post amputation (3dpa) after a second amputation. Scale bars 100 µm. (P) RNAi of putative unspecialized neoblast subset marker, *H3.3*, leads to a loss of major anterior structures, including the nervous system (green) and the pharynx (magenta). Control RNAi showing proper neural expression (*gad-1*) and pharyngeal expression (*pha-1*) in the anterior region of regenerating tail fragments (denoted by white arrowhead). *H3.3* RNAi showing lack of expression of both neural gene expression (green) and pharyngeal gene expression (magenta) in the anterior of regenerating tail fragments (denoted by yellow arrowhead). *H3.3-2* RNAi showing the return of concentrated neural gene expression (green) and pharyngeal gene expression (magenta) in the anterior of regenerating tail fragments (denoted by red arrowhead). Regenerating tail fragments are shown 7 days post amputation (7dpa) after a second amputation. Scale bars 100 µm. (Q) RNAi of putative unspecialized neoblast subset marker, *H3.3*, leads to diminished expression of *piwi-1*$^+$ cells (green) marked by *vax*, *foxF*, and *foxJ1* (magenta) which are associated with neural, muscle and epidermal lineages. Images are taken at the wound site at 72hpa. Scale bars 10 µm. (R) UMAP of merged regeneration neoblast subpopulations showing *FoxO* and *tbx* gene expression. Schematics of regenerating *Hofstenia* fragments in (M-P) reprinted from Cell Reports, Volume 32, Issue 9, Ramirez, A.N., Loubet-Senear, K., & Srivastava, M., A Regulatory Program for Initiation of Wnt Signaling during Posterior Regeneration, 108098, Copyright (2020), with permission from Elsevier.
